# Defects and defect-mediated engineering of two-dimensional materials: challenges and open questions

**DOI:** 10.3762/bjnano.17.31

**Published:** 2026-03-31

**Authors:** Arkady V Krasheninnikov, Matthias Batzill, Anouar-Akacha Delenda, Marija Drndić, Chris Ewels, Katharina J Franke, Mahdi Ghorbani-Asl, Alexander Holleitner, Ado Jorio, Ute Kaiser, Daria Kieczka, Hannu-Pekka Komsa, Jani Kotakoski, Manuel Längle, David Lamprecht, Yun Liu, Steven G Louie, Janina Maultzsch, Thomas Michely, Katherine Milton, Anna Niggas, Hanako Okuno, Joshua A Robinson, Marika Schleberger, Bruno Schuler, Alexander Shluger, Kazu Suenaga, Kristian S Thygesen, Richard A Wilhelm, E Harriet Åhlgren, Carla Bittencourt

**Affiliations:** 1 For addresses of all authors, please see the end of the main text.

**Keywords:** 2D materials, defects, electron irradiation, ion bombardment

## Abstract

Compared to bulk solids, defects in low-dimensional materials and, specifically, 2D systems are expected to have a stronger effect, detrimental or beneficial, on their properties. Owing to their geometry, defects in 2D materials can easily be formed due to the interaction with the environment or under impacts of energetic particles, such as ions and electrons. At the same time, many concepts of defect production under irradiation in bulk systems are not applicable for 2D materials or require substantial modifications. Various aspects of the physics and chemistry of defects in 2D materials have been addressed, and the results of these investigations are presented in hundreds of research papers and review articles. However, the challenges and open questions that still remain in the field have received relatively little attention. These topics were recently addressed at the symposium “Defect-mediated engineering of nanomaterials for energy and quantum applications” organized by the Beilstein-Institut. Following the discussions at the symposium, here, we present the challenges and open questions in our understanding of the behavior of defective 2D materials, interaction of energetic particles with low-dimensional targets, and defect-mediated engineering of the properties of 2D systems. We further discuss possible solutions to these problems or suggest “work-arounds”, which should accelerate the progress in the field.

## Introduction

Defects, which appear in crystalline solids at finite temperatures due to the second law of thermodynamics, are also present in two-dimensional (2D) systems, an important class of materials that have recently received enormous amount of attention due to their unique properties [1]. Moreover, many 2D materials are synthetic; hence, in principle, the defect concentration in them can be well above the equilibrium value as the time after their fabrication may not have been sufficient to reach equilibrium. 2D materials have a high surface-to-volume ratio, so that defects can easily be formed due to the interaction with the environment, for example, because of oxidation. The imperfections have a strong influence on the electronic, optical, thermal, and mechanical properties of 2D materials [2–3]. They normally deteriorate the characteristics; but they can also be beneficial, for example, in the context of doping or single-photon quantum emitters [4–5].

Moreover, irradiation of 2D materials with energetic particles, that is, ions and electrons, has been demonstrated to be a suitable approach for tailoring their properties through the controllable creation of defects and the introduction of impurities, see [6–10] for an overview. Specifically, low-energy ion implantation [11–17] can be employed to directly create impurities in 2D materials. Alternatively, due to the 2D geometry, impurities can be introduced through filling the vacancies previously created by impacts of energetic particles [18–21] by foreign atoms.

Lots of insights into the structure and properties of defective 2D materials have been obtained using transmission electron microscopy (TEM), including conventional TEM and scanning TEM (STEM). These techniques, along with scanning probe methods, can provide information on the atomic structure of materials with sub-angstrom resolution [22]. The aberration-corrected TEM has made it possible not only to obtain images of impurities [23–26] and intrinsic point defects [27–31], but also to follow their evolution in real time. However, it was realized long ago [32–33] that energetic electrons in the TEM interacting with the specimen can give rise to the formation of defects or even to the complete destruction of the sample, an undesirable effect, which stimulated the research on beam–sample interactions and the mechanisms of defect formation. This indicates that defects can also be created deliberately during exposure to the electron beam, which can be used for engineering structure and properties of materials with potentially atomic resolution [34–37].

It should be pointed out that many concepts of defect production under irradiation in bulk systems are not applicable for 2D materials or require substantial modifications. Various aspects of the physics and chemistry of defects in 2D materials have been addressed; based on the results of these investigations, hundreds, if not thousands, of papers have been published. The findings have been summarized in several review articles [2–3,6–10]. However, the challenges and open questions that still remain in the field have received relatively little attention.

In May 2025, these topics were addressed at the symposium “Defect-mediated engineering of nanomaterials for energy and quantum applications”, organized in Rüdesheim, Germany, by the Beilstein-Institut [38]. Following the symposium, in this article, we discuss the challenges and open questions, in our understanding, of the behavior of defective 2D materials, interaction of energetic particles with low-dimensional targets, and defect-mediated engineering of the properties of 2D materials.

## Challenges and Open Questions

### Can the defect concentration in epitaxial 2D materials be as low as in 2D materials exfoliated from their bulk counterparts?

In bulk materials, generally, the lower defect formation energies at the surfaces result in much higher surface defect concentration compared to the bulk. This gives rise to the following questions: Is this also true for van der Waals materials? Do thermodynamic considerations in transition metal dichalcogenides (TMDs) with relatively low (compared to graphene or h-BN) defect formation energies prevent low defect concentrations in monolayer sheets? How can synthesis methods for monolayer materials be modified to control defect species and concentration? We note that, in TMDs, not only chalcogen vacancies, but also transition metal vacancies and antisites, have been reported. In synthetic materials, the defect concentration depends on the growth conditions; however, these are rarely systematically studied and, if so, only over fairly narrow accessible ranges of parameters, for example, growth temperatures. Moreover, there are large variations between different growth methods, for example, chemical vapor transport vs molecular beam epitaxy in terms of pressures and growth temperatures. Generally, the thermodynamic conditions are ill-defined during growth, especially the chemical potentials of the material constituents are not well known, making a comparison to the theory difficult.

A possible solution is to use a different strategy: For systematic studies of the thermodynamic conditions (background pressure and temperature), synthesis systems need to be developed that allow to vary these conditions (partial pressures of the constituents and temperature) over a wide range. An alternative, and possibly more accessible for monolayer materials, is to control defect concentrations by post-growth processing in controlled atmosphere of chalcogens (or other volatile components of 2D materials) and temperatures. Equilibration of the gas phase chemical potential with 2D materials determines the defect concentration. Ideally, in situ measurements of defects are employed, but given the challenging atmosphere this may require optical methods, which may not have the required sensitivity to small defect concentrations. Ex situ microscopy measurements (scanning tunneling microscopy (STM) and STEM) are slower and may introduce uncertainties due to the required change in the thermodynamic conditions during transfer from the processing chamber to the vacuum in the microscope.

### Are defect complexes in sub-stoichiometric/impurity-doped 2D materials more frequent than in bulk materials? Can complex multiatom defects form extended/periodic structures in 2D systems?

The reduced dimension of 2D materials enables easier structural relaxations and fewer bond “rearrangements” compared to 3D covalently bonded materials. This may affect defect formation energies and also enable the formation of complex defect structures in sub-stoichiometric or impurity-doped materials. Examples are borophene [39] due to its polymorphism [40] and mirror twin boundaries (MTBs) and MTB-networks in non-stoichiometric MoSe_2−_*_x_* or MoTe_2−_*_x_* due to lower formation energies of such structures compared to those for isolated vacancies [41]. The complexity of defects and the large number of systems means that the discovery of such new materials relied mostly on “accidental” experimental observations. Under most synthesis conditions, the phases with the lowest formation enthalpy are formed for a given starting composition mixture. If the composition is determined by the element mixture (e.g., if the elements are sealed in a reactor or ampule) the system may separate into compositional phases that minimize the overall formation enthalpy. Materials with intermediate stoichiometries are difficult to obtain. Similarly, in thin film growth involving volatile components (e.g., chalcogens), the composition of the film is determined by the compound with the lowest formation enthalpy and not by the atom flux. For example, during molecular beam epitaxy (MBE) growth of chalcogenides, chalcogen atoms are generally supplied in excess, and the film will adopt the lowest-energy composition. In these cases, the stoichiometry of the film is not determined by the composition of the source materials but by the thermodynamic stability of the film. So again, in thin film growth, obtaining compositions that are not close to the lowest-energy compound is challenging. This is also true for incorporation of impurities at higher concentrations.

Again, a possible solution is to use a different strategy: 2D materials offer a unique solution to the design of materials with different compositions and the formation of metastable defect complexes with sub-stoichiometric compositions, namely, the post-synthesis reaction of the 2D material with an element that is desired to be incorporated. This may be accomplished by modifying 2D sheets with vapor-deposited atoms, for example, the reaction of MoSe_2_ with excess Mo to form MTB networks [42]. Such surface reactions can change the composition and induce substoichiometric defects. The approach of reacting 2D sheets (e.g., chalcogenides) with excess metal may result in metastable point- and line-defect configurations [43–45] and even dense line-defect networks [46–48]; it can be further extended to reacting with heteroatoms to introduce electronic dopants, magnetic impurities, and catalytic sites, as well as to add other functionalities [43,49–50]. However, challenges remain to have reliable predictions for the outcome of the reaction and to go beyond a “trial and error” approach.

### What are the oxidation mechanisms and kinetics of defective surfaces in 2D materials?

Substitutional oxygen defects are present in 2D materials synthesized via chemical vapor deposition (CVD) [51]. This can be a result of oxide precursors, which are frequently used. Moreover, 2D materials are known to oxidize when exposed to oxygen gas and water [52–53]. The reaction of molecular oxygen with the surfaces of 2D materials has attracted significant interest [54]. The reaction barrier for O_2_ dissociation on a TMD surface is almost halved comparing a pristine surface with one containing a sulfur vacancy, decreasing from 1.59 to 0.80 eV [55–58]. This suggests that the reaction of O_2_ with a pristine surface is unlikely [59–60] and that S vacancies are likely to play an important role in the process.

STM measurements on MoS_2_ demonstrated a high intrinsic concentration of individual S vacancies and a lower concentration of S vacancy dimers in exfoliated single-layer films [61]. In contrast, density functional theory (DFT) calculations reveal that molecular oxygen reacts very readily with larger S vacancy clusters (three or more vacancies), with O_2_ dissociation being highly exothermic and proceeding with small or negligible reaction barriers [54,62]. Such low barriers imply that, once formed, larger vacancy clusters can promote a near-instantaneous reaction with O_2_. However, the formation of these clusters cannot be explained by vacancy diffusion, as the migration barriers for S vacancies on the WS_2_ surface exceed 2 eV, rendering vacancy migration unlikely at room temperature and under typical experimental conditions [62]. This suggests that large vacancy clusters form as a result of non-diffusive processes, such as sputtering and etching of the surface.

**Figure 1:**
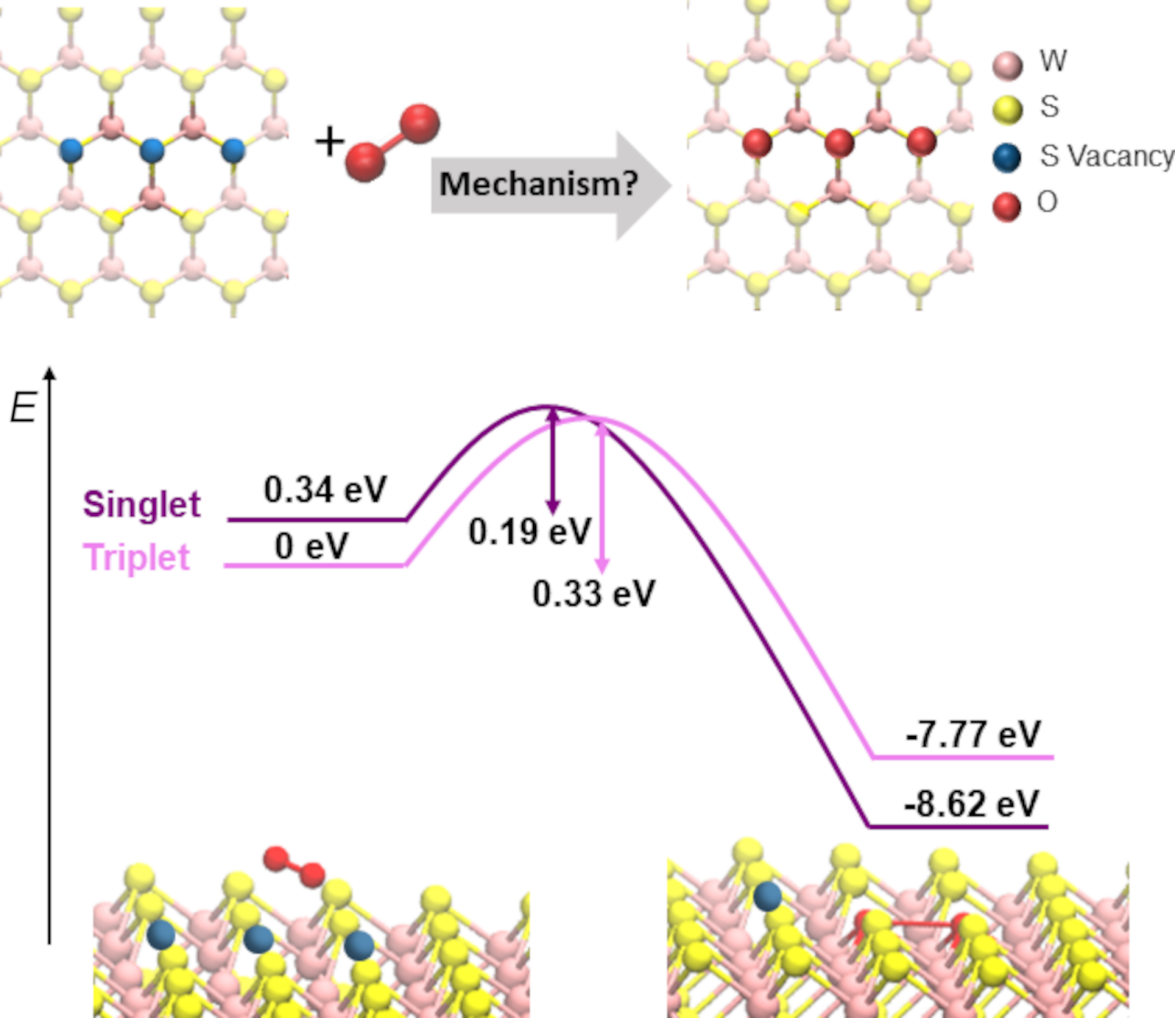
Schematic of the reaction of an O_2_ molecule with a sulfur trivacancy on the surface of WS_2_. Upper and lower part of Figure 1 was adapted from [62] (“Unveiling surface dynamics: in situ oxidation of defective WS_2_”, © 2025 D. Kieczka et al., published by the Royal Society of Chemistry, distributed under the terms of the Creative Commons Attribution 3.0 Unported Licence, http://creativecommons.org/licenses/by/3.0/).

Despite the high reactivity of larger S vacancy clusters, several challenges remain in describing the oxidation kinetics. O_2_ is bound weakly to TMD surfaces with a binding energy of ≈0.14 eV [55,63–65]. Ab initio molecular dynamics (MD) simulations show that O_2_ is therefore mobile on the pristine surface of WS_2_, resulting in rapid desorption from the surface at room temperature. The short residence time limits encounters with reactive defect sites, leading to a small effective reaction cross section for vacancy dimers and trimers; dissociation requires overcoming a finite barrier (see Figure 1), reducing the likelihood of O_2_ reactions with these defect sites. Reactivity is also highly site-specific and controlled by the local coordination and electronic structure of surface W atoms. The effective charge of W is affected by the number of surrounding vacancies. W atoms surrounded by three or more S vacancies exhibit reduced effective charges and are more chemically active. This enables barrier-less reactions of O_2_, explaining the rapid oxidation at larger vacancy clusters.

Additional uncertainty in modelling oxidation kinetics arises from the possibility of changes in the spin state of the O_2_ molecule from triplet to singlet during dissociation [62]. Although O_2_ incorporation into the WS_2_ surface involves electron transfer from the substrate, the reaction may proceed via non-adiabatic pathways, as previously discussed for O_2_ adsorption on metal surfaces [66–67]. This underscores the limitations of purely adiabatic descriptions. The mechanism of this process is still poorly understood and requires more detailed studies, particularly on surfaces of semiconductors.

Another factor that affects the mechanism and kinetics of oxidation is the exothermicity of the reaction. The O_2_ incorporation into S vacancy clusters is highly exothermic and should be accompanied by dissipation of about 8.6 eV on the WS_2_ surface [62]. Modelling the mechanism of this dissipation is a complex problem. In addition to creating heat and electron–hole pairs, it may involve desorption of atoms and molecules, such as SO_2_ [60], which can propagate further the formation of larger vacancy clusters. It is expected that the mechanisms of oxidation will be similar for multilayer films and defective monolayers and driven by the effective charges of W atoms exposed by S vacancy clusters. However, changes in the positions of the valence band maximum and conduction band minimum between bulk and monolayer WS_2_ affect the relative position of the O_2_ LUMO responsible for the charge transfer. This can affect the barrier of O_2_ dissociation and requires further studies.

The mechanisms of propagation of oxidation front from the surface of multilayer films into the bulk are poorly understood. In relatively unstable TMDs, such as MoTe_2_ and WTe_2_, an ultrathin amorphous layer of MoO_3_ or TeO_2_ is immediately formed when CVD or MBE-grown MoTe_2_ is exposed to ambient environments [53,68]. However, the barrier for S vacancy diffusion between TMD layers was predicted to be very high [62], hampering the vacancy diffusion mechanism. Nevertheless, a relatively thick native oxide layer can still form on otherwise stable HfS_2_ after plasma oxidation. In this process, O ions are believed to permeate through grain boundaries between small crystals and react with HfS_2_ beneath the surface [69]. The mechanisms and kinetics of such processes are still poorly understood.

At the same time, the edges of MX_2_ nanoribbons exhibit strong driving forces for O_2_ dissociation and chemisorption, where 100% metal edges have the highest rates of oxidation [56,70]. In addition, previous studies have demonstrated that H_2_O can significantly accelerate the oxidation of TMDs, with the formation of sulfuric acid and metal oxide complexes reported [71–72]. The interactions and reactions of O_2_ and H_2_O at defects sites and edges that initiate and propagate oxidation in TMDs still require further study. Moreover, it has been shown that even the exposure of TMD films to ordinary laboratory light in air can accelerate oxidation [73], although the underlying mechanism of this effect remains unclear.

### Does high-temperature annealing increase or decrease the concentration of defects in 2D materials?

In bulk systems, when the defect concentration *n* is well above the equilibrium value *n*_eq_ at temperature *T*, defined by the defect formation energy *E*_f_,


[1] $$n_{\text{eq}}=\exp\left(-E_{\text{f}}/k_{\text{B}}T\right),$$

*n* normally decreases upon high-temperature annealing in vacuum, as defects migrate towards the surface and disappear. The evaporation of the material at the surface is ignored. The situation is different in 2D materials [74] as the concentration of intrinsic defects or impurities (e.g., oxygen in MoS_2_) can decrease at moderate-temperature annealing [75], but ablation of atoms at high temperatures cannot be ignored, and more vacancies can appear. At the same time, annealing always removes (at least partially, when agglomeration of material occurs) adatoms and organic molecules from the surface of a 2D system, which can also be referred to as defects. The optimum annealing temperature depends on the sublimation energy and is material-dependent.

A counterintuitive approach to decrease the concentration of surface defects in layered TMDs was also suggested [76], namely, a two-step process including low-energy (500 eV) Ar ion bombardment followed by annealing. It was demonstrated that the concentration of Te vacancies on the as-cleaved PtTe_2_ and PtTe_2_ surfaces can be decreased by more than 99%, which cannot be achieved by annealing alone. The experimental observations were rationalized through the enhanced diffusion of atoms in the irradiated samples.

However, annealing in a specific atmosphere can give rise to a drop in the concentration of intrinsic defects; for example, annealing in S atmosphere should decrease the number of S vacancies in MoS_2_. The geometry of 2D materials also makes it possible to introduce impurities upon annealing in a specific atmosphere or to create new 2D systems; a good example is the conversion of 2D MoSe_2_ on a gold substrate to the Janus MoSeS structure [77]. Also, a facile low-temperature thiol chemistry route was suggested to remove sulfur vacancies in 2D MoS_2_ [78], which resulted in a significant drop in the number of the charged impurities and traps and increased the mobility. Qualitatively similar results were also reported after treatment with ethylenediaminetetraacetic acid [79].

### Which techniques can be used to assess defect concentration in 2D materials? What are the challenges for each technique?

Defect concentration can be assessed by techniques that allow for the direct visualization of defects, for example, TEM [80], STM [81], or atomic force microscopy (AFM) [82]; but, normally, only a small part of the sample can be probed [83–85]. Moreover, these techniques can create defects themselves, for example, when the energy of the electron beam exceeds the displacement threshold for the atoms in the investigated material, or under specific imaging conditions using STM, as described in section “Where are the limits for controlled defect engineering in terms of spatial precision, single type or size (e.g., hole size), and scalability?”.

**Figure 2:**
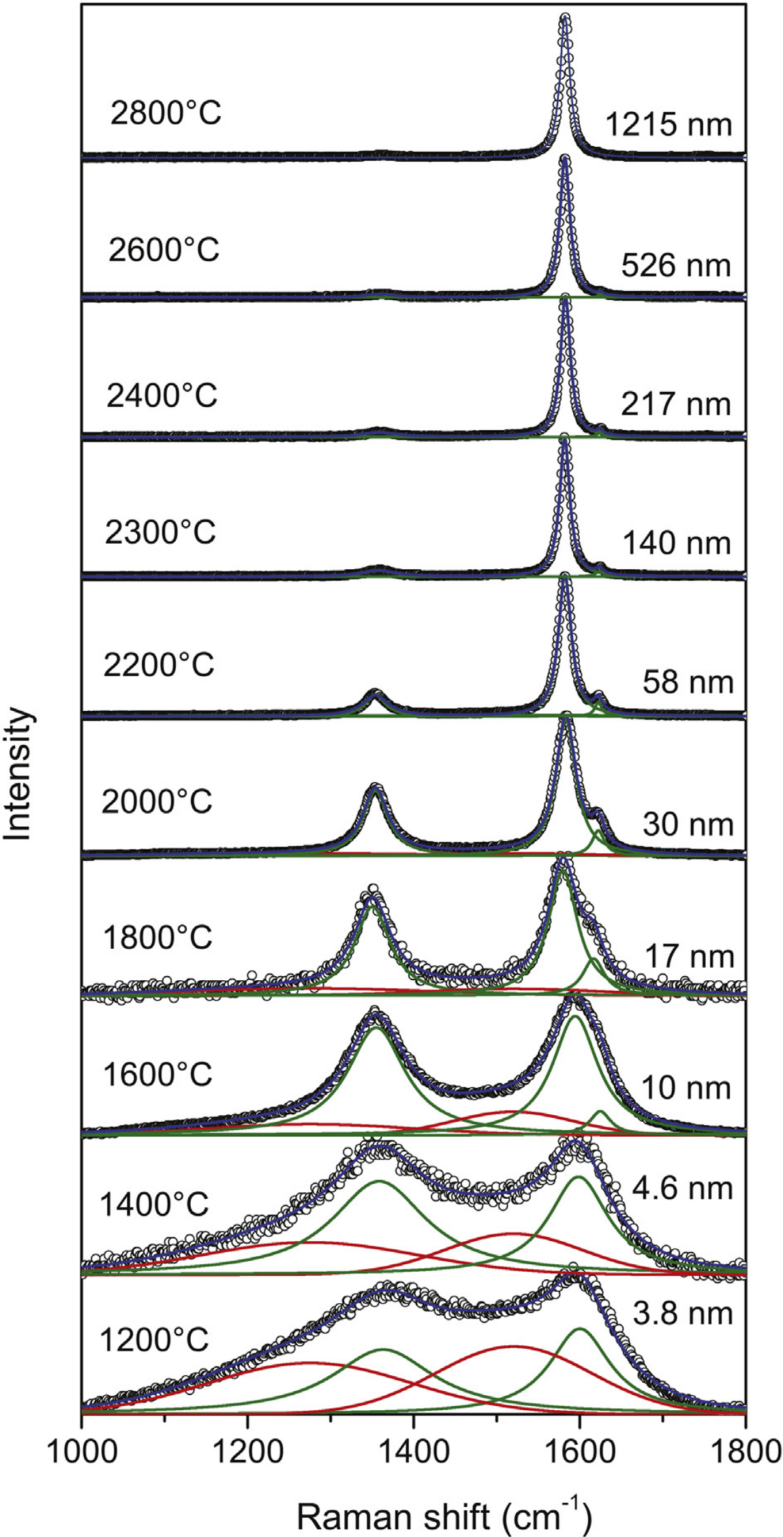
Raman spectra of graphene samples produced by heat treatment of diamond-like amorphous carbon (DLC) [87–88], which is known to produce graphite nanocrystallites with lateral dimension (*L_a_*) defined by the heat treatment temperature (HTT) [86]. The HTT and *L_a_* (obtained by X-ray diffraction) are indicated on the left and right sides of each spectrum, respectively [88]. Figure 2 was reprinted from [88], *Carbon*, Vol. 95, by J. Ribeiro-Soares, M. E. Oliveros, C. Garin, M. V. David, L. G. P. Martins, C. A. Almeida, E. H. Martins-Ferreira, K. Takai, T. Enoki, R. Magalhães-Paniago, A. Malachias, A. Jorio, B. S. Archanjo, C. A. Achete, L. G. Cançado, “Structural analysis of polycrystalline graphene systems by Raman spectroscopy”, Pages 646–652, Copyright (2015), with permission from Elsevier. This content is not subject to CC BY 4.0.

As a non-invasive technique, Raman spectroscopy has led to metrological procedures to access defect concentration in graphene. Well-established formulae are available to determine defect quantity based on the D/G ratio and the linewidth of the G band (see Figure 2) [86–89].

In a wider context, while Raman-active local vibrational modes can, in principle, emerge due to defects [90–91], in practice they are rarely observed owing to the low concentration of defects and low signal-to-noise ratio, which becomes particularly pertinent with 2D materials due the small volume for scattering. In most cases, one observes changes in the Raman-active modes of the host material regarding peak position, lineshape, and the relative intensities of peaks. The position and relative intensities are affected by strain and doping induced by defects. In addition, relaxation of the translation symmetry leads to activation of modes that would otherwise be forbidden. At low defect densities, this leads to asymmetric line broadening, where the asymmetry reflects the frequency-dependence of the mode around the Γ point. At high defect densities, modes across the whole Brillouin zone can be activated [91–93]. This naturally limits the distinguishability among defects as several defects can yield similar strain, doping, or mode activation. In principle, defects might be separated by comparing the effects on all observed peaks [94]. Unfortunately, many common 2D materials only show a few Raman-active peaks under non-resonant conditions; this problem can be somewhat alleviated by doing the measurements under resonance conditions, which leads to activation of a large number of peaks.

Another major issue is that one cannot directly read out the identity of a defect from the spectrum; instead, comparison to reference spectra is required. Such information is rarely available experimentally, owing to difficulties in creating on demand a single type of defect of known concentration. However, type and concentration of defects can be exactly defined in calculations, which is promising for producing reference data. In the past, such calculations have been computationally prohibitively costly, but modern machine learning models have enabled such simulations for non-resonant Raman experiments.

**Figure 3:**
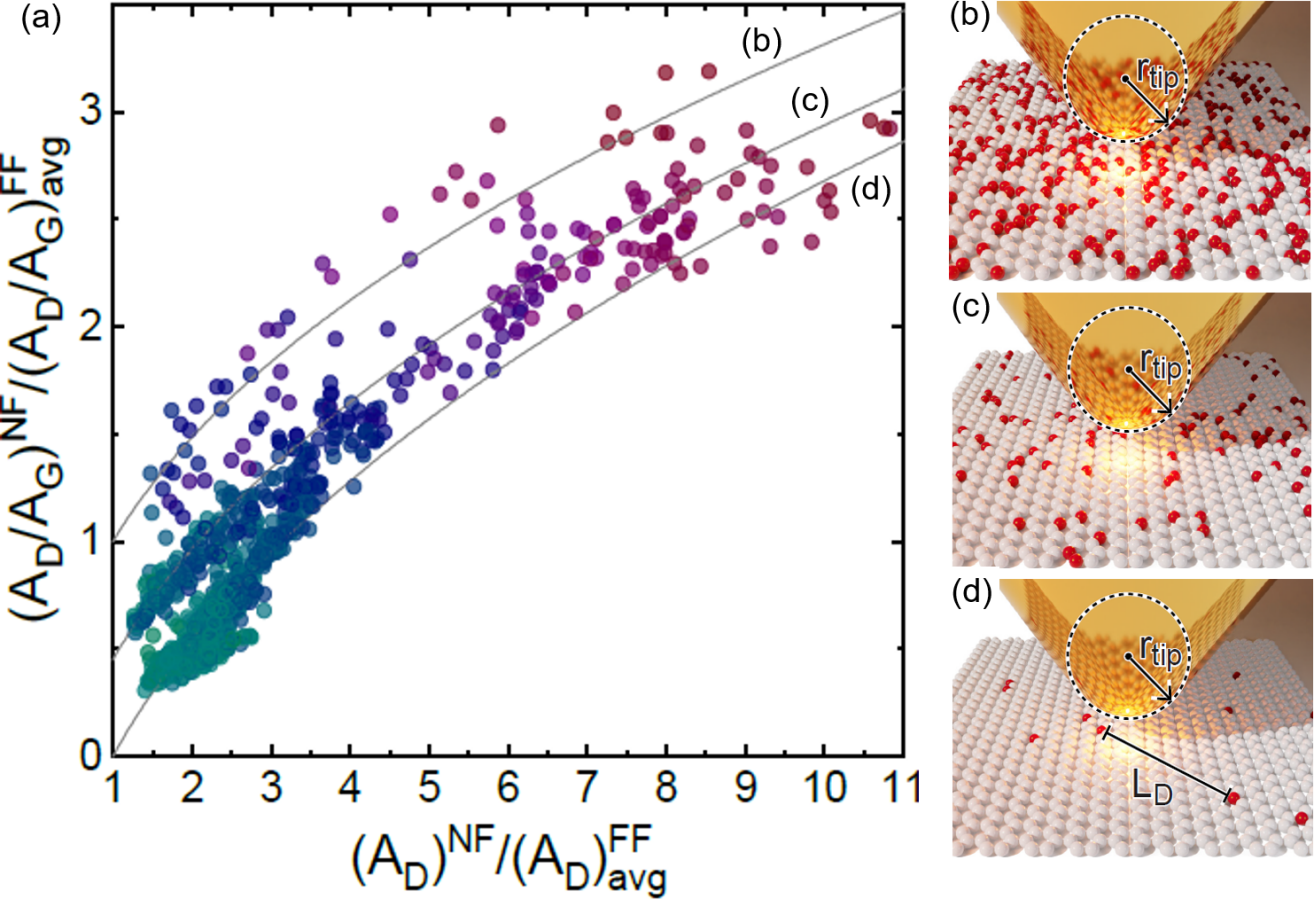
(a) Spatial distribution of the D/G ratio obtained on graphene with nano-Raman spectroscopy as a function of the local D-band enhancement. The local peak areas *A*^NF^ are normalized by the averaged micro-Raman areas *A*^FF^. Panels (b–d) are schematics showing the TERS tip (yellow) on top of graphene (white) with point defects (red), in the limits (b) *L*_D_ *< r*_tip_, (c) *L*_D_ ≈ *r*_tip_, and (d) *L*_D_ *> r*_tip_, where *L*_D_ is the average distance between defects, and *r*_tip_ the tip apex radius [97]. Figure 3 was used with permission from [97] (“Nano-Raman spectroscopy of 2D materials”, by A. Jorio et al., *2D Materials*, Vol. 11, Number 3, Article No. 033003, published on 14 May 2024; https://doi.org/10.1088/2053-1583/ad42ad); © 2024 IOP Publishing Ltd; permission conveyed through Copyright Clearance Center, Inc. All rights reserved. This content is not subject to CC BY 4.0.

Therefore, the great challenge is the development of higher-precision measurements utilizing nano-Raman spectroscopy (see Figure 3). When nano-Raman is implemented in the tip-enhanced Raman spectroscopy (TERS) configuration, the well-established D/G ratio depends on the TERS tip enhancement [95] and whether the distance among defects is larger or smaller than the tip apex size [96–97]. As shown in Figure 3, the D/G ratio exhibits three different tendencies depending on the relative dimension between the TERS probe (*r*_tip_ is the tip apex radius) and the distance among defects (*L*_D_) [97].

### Raman spectroscopy has been used to estimate defect concentrations in graphene. How does it work for other 2D materials?

Several authors have been addressing the use of Raman spectroscopy to quantify defects in TMDs [93,98–102]. However, while, in graphene, defects give rise to new peaks in the spectrum (e.g., the so-called D band), as introduced in the 1970s by Tuinstra and Koenig [103] and further developed into metrological procedures (see Figure 2) [86–89,104], imperfections do not drastically change the Raman spectra of other 2D materials. Normally, the peaks are broadened, and their relative position may change as, for example, in MoS_2_ [105].

**Figure 4:**
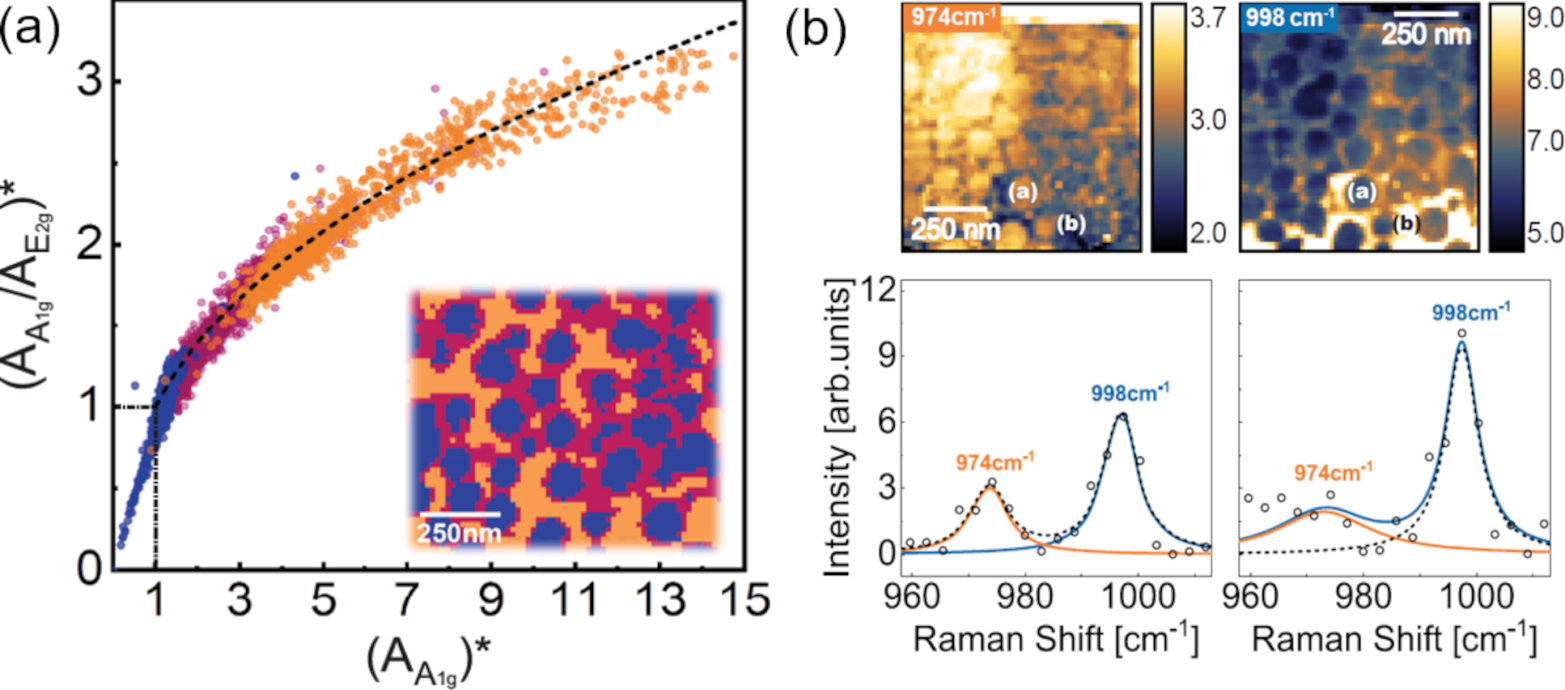
(a) Spatial distribution of the *A*_1g_/*E*_2g_ peak ratio measured on MoSe_2_ with nano-Raman spectroscopy as a function of the local *A*_1g_ peak enhancement. The local peak areas *A*^NF^ are normalized to the averaged micro-Raman areas *A*^FF^. The colors indicate the locations from where the spectral information comes on MoSe_2_, evidencing a dependence on the presence of nano-protuberances due to environmental contaminations. (b) nano-Raman allows one to identify peaks specific to MoO_3_, indicating local oxidation [109]. Figure 4 was adapted from [109] (© 2025 J. E. Guimarães et al., published by American Chemical Society, distributed under the terms of the Creative Commons Attribution 4.0 International License, https://creativecommons.org/licenses/by/4.0).

Extending the higher-precision measurements utilizing nano-Raman spectroscopy developed for graphene (described in the section before) for other 2D systems is a challenge. The advantages of graphene are the high Raman frequencies, the simplicity of the spectra, and the electronic resonance conditions, which are not always as favorable in the case of other 2D materials. However, several authors are addressing this issue with micro-Raman [93,99–102] and nano-Raman [97,106–109] spectroscopies. Experiments performed on MoSe_2_ (Figure 4) show that the dependence observed in Figure 3 for graphene also applies to other 2D materials and that TERS can be utilized to identify specific types of molecular contaminations [109].

### Can Raman spectroscopy be used to not only assess defect concentration but also to differentiate between different kinds of defects?

**Figure 5:**
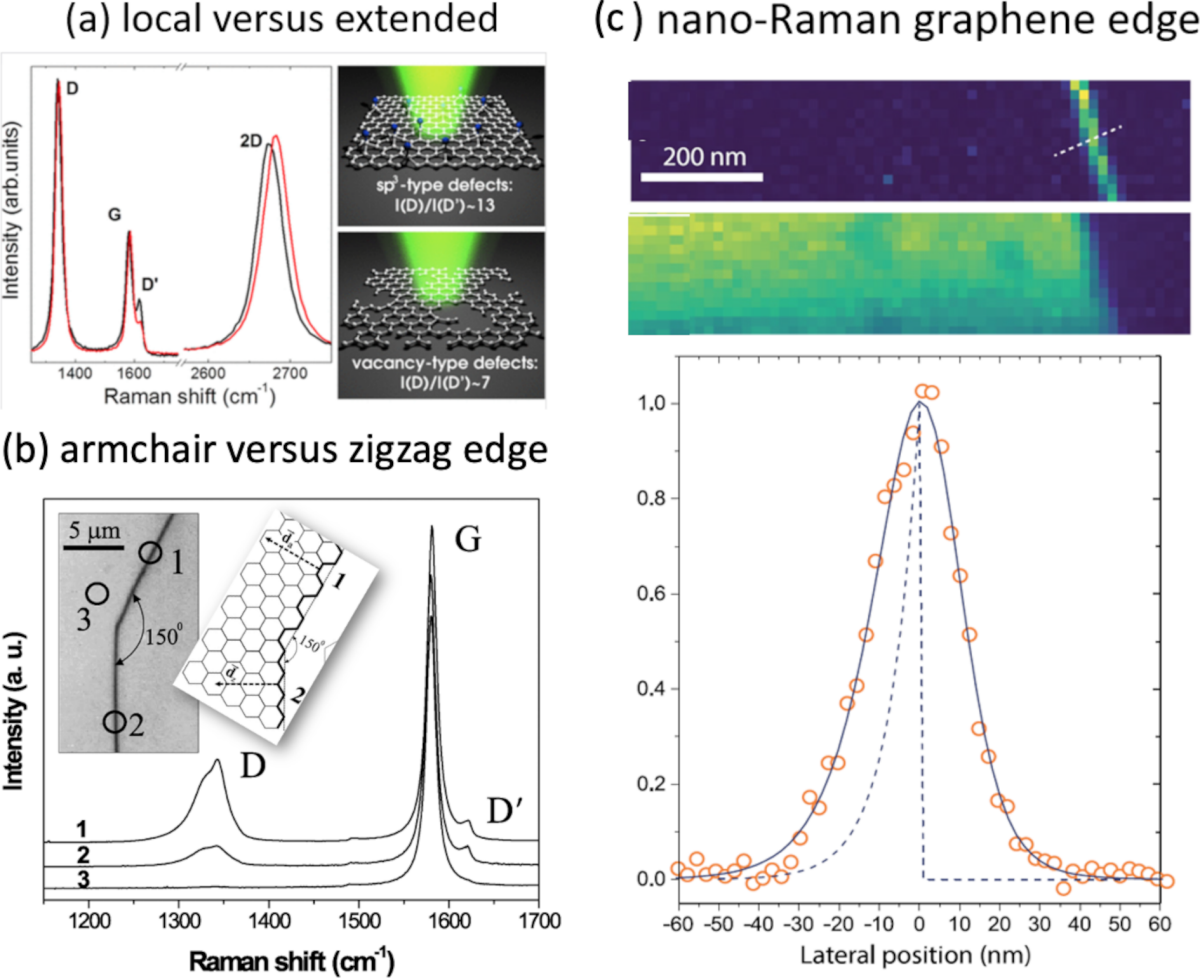
Types of defects in graphene, where Raman spectroscopy has been shown to be able to identify and differentiate defects. (a) The relative intensity of the D′ peak differentiates defects with local versus extended changes in the local potential [110]; (b) the relative intensity of the D peak indicates whether the graphene edge is of zigzag or armchair type [112]; (c) potential deformation of the graphene edge measured by nano-Raman (TERS) [113]. Figure 5a was reprinted with permission from [110]. Copyright 2012 American Chemical Society. This content is not subject to CC BY 4.0. Figure 5b was reprinted with permission from [112], Copyright 2004 by the American Physical Society. This content is not subject to CC BY 4.0. Figure 5c was used with permission from [113] (“Establishing the excitation field in tip-enhanced Raman spectroscopy to study nanostructures within two-dimensional systems”, by H. Miranda et al., *2D Materials*, Vol. 10, Number 1, Article No. 015002, published on 20 October 2022; https://doi.org/10.1088/2053-1583/ac988f); © 2022 IOP Publishing Ltd; permission conveyed through Copyright Clearance Center, Inc. All rights reserved. This content is not subject to CC BY 4.0.

Raman spectroscopy can be utilized to differentiate types of defects in 2D systems. As illustrated in Figure 5 for the case of graphene, local versus extended defect-induced potential [110], *p* versus *n* substitutional doping atoms, [111], and armchair versus zigzag edges [112] can be differentiated.

**Figure 6:**
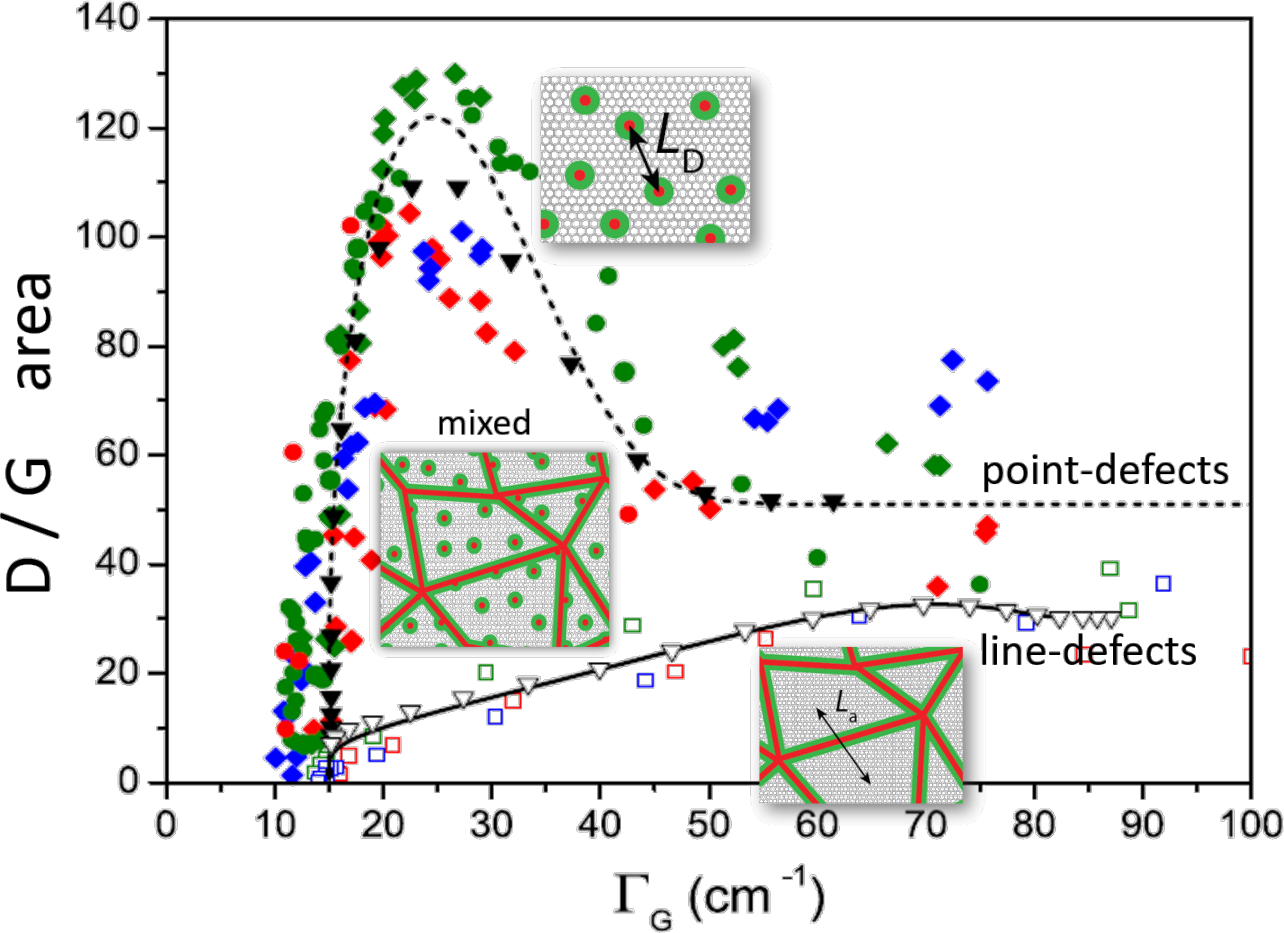
Raman phase diagram to disentangle and quantify the amount of point (0D) and line (1D) defects in graphene. Filled symbols are experimental data from ion-bombarded graphene and open symbols are from graphitized DLC treated at different annealing temperatures [114]. Figure 6 was adapted from [114] (“Disentangling contributions of point and line defects in the Raman spectra of graphene-related materials”, © 2017 L. G. Cançado et al., published by IOP Publishing Ltd, distributed under the terms of the Creative Commons Attribution 3.0 Licence, http://creativecommons.org/licenses/by/3.0).

A fundamental characterization enabled by Raman spectroscopy was the distinction between defect dimensionalities. Geometrically, a 2D system can have zero-dimensional (0D) defects and one-dimensional (1D) defects. 0D are point defects, like a vacancy or a substitutional atom, while 1D are line defects, like crystallite borders or mirror twin boundaries. Based on the geometrical model introduced in [81], it was possible to determine a phase diagram based on the D/G ratio and the G band linewidth to disentangle and quantify the amount of point and line defects in graphene (Figure 6) [114]. We have not achieved, so far, the capability to image the atomic structure of the defects with Raman spectroscopy. Even nano-Raman still lacks resolution for that [113] (Figure 5c).

### What are the challenges in the calculations of Raman spectra of defective 2D materials?

In order to correctly describe how the phonons of the pristine material are affected by defects, calculations of Raman spectra require large supercells to describe the low defect density and their random distribution, which makes it computationally extremely demanding to determine structural dynamics, whether from molecular dynamics or from harmonic approximation plus finite displacements. This can nowadays be overcome by using machine learning interatomic potentials (MLIPs), mostly for pristine materials, but in a few cases also with defects [94,115].

In order to model Raman scattering, a model for the response of the material to incoming light is required. In the non-resonant case, Raman scattering is allowed when the atomic displacement of a vibrational mode leads to a (first-order) change in the electronic susceptibility (or polarizability). Similar to machine learning (ML) models for energies, one can also build ML models for polarizability [116–118]. The main difference is that Raman scattering is described by a second-rank tensor, which depends on the polarizations of the incoming and the scattered light. In addition, first-principles calculations of polarizability are significantly more demanding than those of total energy, which is especially true for large systems with defects; this can limit the collection of training data. Finally, training a model for resonant Raman is much more demanding as it should correctly describe the changes in the energy of electronic states and the transition dipoles. In addition, the largest resonance effects are usually seen at energies where polarizability changes quickly, that is, where it is most sensitive to the atomic displacements. This enhances the difficulty of creating accurate models. Consequently, while machine learning models for resonant Raman have been explored for pristine materials [117], to the best of our knowledge this has not been attempted for defective systems.

### Can optical spectra from individual defects be measured at the atomic scale and correlated with the local atomic and electronic structure?

The ability to measure the optical spectra of individual defects at the atomic scale and to correlate them directly with their local atomic and electronic structure, as performed for 1D carbon nanotubes [111], would mark a decisive advance in the study of 2D semiconductors. Defects in TMDs and h-BN host localized excitonic states and robust single-photon emission [119], yet, their spectroscopic variability has remained enigmatic. Far-field optical probes have revealed striking spatial heterogeneity in emission energies, linewidths, and photon correlations, underscoring the importance of local strain, dielectric fluctuations, moiré potentials, and defect proximity. However, the mismatch between atomic-scale lattice-resolving techniques and micrometer-scale far-field optical spectroscopy has long obstructed unambiguous identification of the emitters’ microscopic nature.

Scanning tunneling microscopy luminescence (STML) offers a compelling solution. By combining atomically resolved imaging and spectroscopy by means of scanning tunneling microscopy/scanning tunneling spectroscopy (STM/STS) with localized electrically driven light emission in the STM junction, STML can, in principle, assign the optical fingerprint of a single defect directly to its atomic configuration and electronic states. Such a capability would allow the emission characteristics of defect-bound or moiré-trapped excitons to be traced with sub-nanometer precision, while also probing vibronic structure and photon statistics in situ. Complementary approaches, such as tip-enhanced photoluminescence, extend this toolbox by enabling nanoscale hyperspectral mapping and photon correlation without charge injection. Together, these techniques promise to recover the full optical signatures of individual emitters, to verify quantum emission directly at their atomic site, and to reveal how specific structural motifs dictate their photophysics.

### Can we establish a comprehensive database of defect fingerprints for defect identification/quantification using STM/STS?

**Figure 7:**
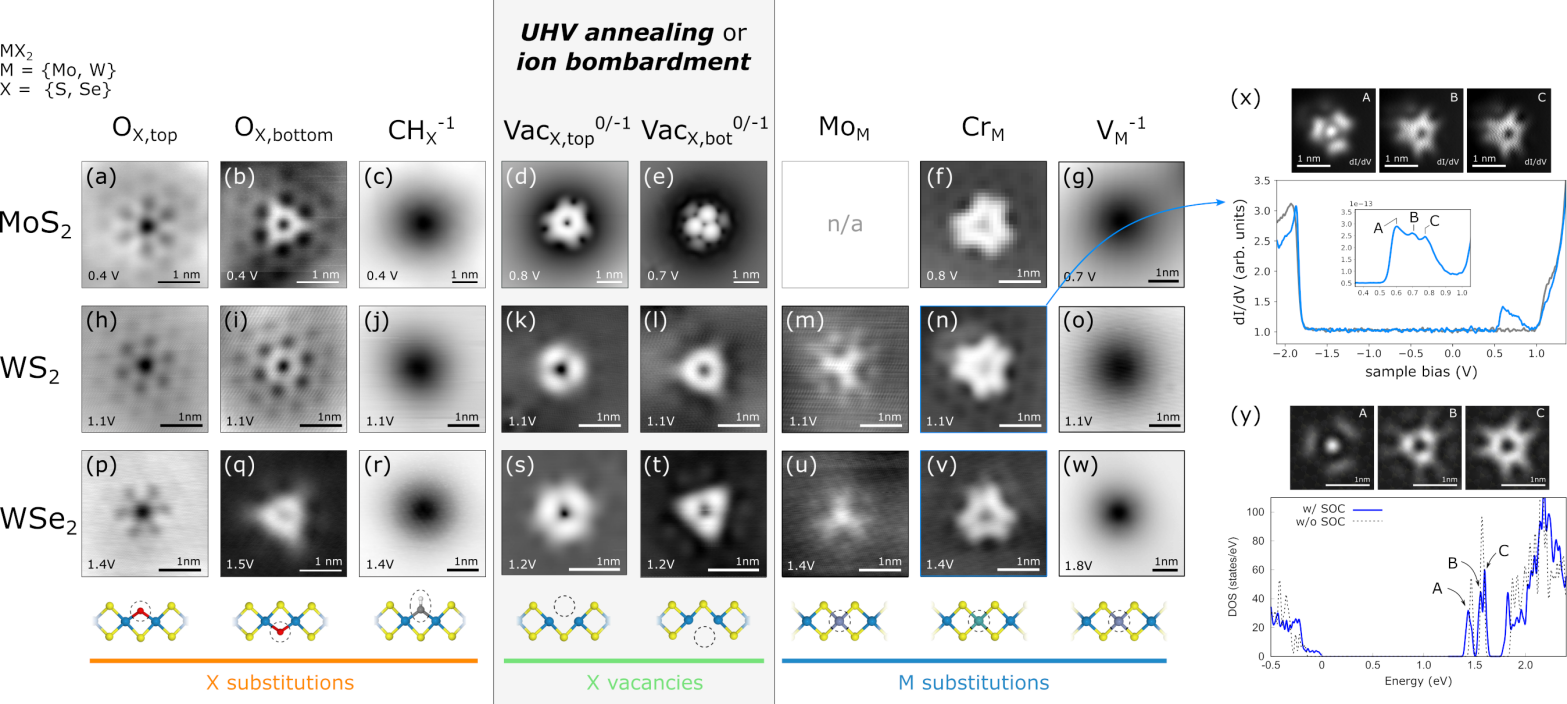
Overview of STM images of common point defects in synthetically grown TMDs. Panels (a–g) show defects in MoS_2_, panels (h–o) and (x, y) show defects in WS_2_, panels (p–w) show defects in WSe_2_. Panels (a–c), (f, g), (p–r), and (u, v) present unpublished STM data acquired by B. Schuler. Panels (d, e) were adapted from [127] (© 2024 by F. Xiang et al. published by Springer Nature, distributed under the terms of the Creative Commons Attribution 4.0 International Licence, http://creativecommons.org/licenses/by/4.0/). Panels (h–o) and (x, y) were adapted with permission from [120]. Copyright 2019 American Chemical Society. This content is not subject to CC BY 4.0. Panels (s, t) were adapted from [131] (“Layer-Dependent Charge-State Lifetime of Single Se Vacancies in WSe_2_”, © 2025 by L. Bobzien et al. published by the American Physical Society, distributed under the terms of the Creative Commons Attribution 4.0 Licence, http://creativecommons.org/licenses/by/4.0/). Panel (w) was adapted with permission from [123]. Copyright 2023 American Chemical Society. This content is not subject to CC BY 4.0.

STS and orbital imaging provides unique fingerprints of point defects in TMDs including common impurities found in synthetically grown TMDs, such as oxygen substitutions (O_X_) [51,120] and carbon–hydrogen complexes (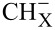) [121–122] at chalcogen sites, molybednum (Mo_M_) [120], chromium (Cr_M_) [120], and vanadium (V_M_) [123] at the transition metal site, as well as annealing or ion bombardment-induced chalcogen vacancies (Vac_X_) [124–127]. In Figure 7, STM topographies of common defects in TMDs recorded close to the conduction band edge are shown. These reference data can be used to assign defects in other samples to allow for a non-invasive metrology standard. More data is available for deliberately doped TMD samples [128] and Pt chalcogenides [129–130]. Nevertheless, a comprehensive data base of the STM images of defects for different bias voltages and corresponding spectra is still to be developed.

### How can defect engineering be used to tune electronic properties of 2D materials? Do shallow defect states exist in 2D semiconductor materials? What is the origin of n-type doping in MoS_2_ and p-type doping in WSe_2_?

Shallow dopants have energy levels close to the band edges; hence, a significant portion of the carriers can be ionized under operating conditions, that is, the ionization energy should be similar in magnitude to *kT*. In conventional 3D semiconductors, substitution of host atoms by those from the neighboring columns of the periodic table often results in shallow dopants. These can be modelled by the effective-mass hydrogenic model (essentially the Schrödinger equation for the hydrogen atom, but using the effective mass of the band edges and screening of Coulomb interaction by the dielectric constant), which yields not only a small ionization energy, but also a highly extended defect state wave function.

The same strategy appears somewhat ineffective in 2D semiconductors. Let us consider substitutional Re@Mo and Nb@Mo defects in MoS_2_. Already in bulk MoS_2_, the wave function is largely confined within a single layer. Confining the hydrogenic effective-mass equation from 3D to 2D shows that the ionization energy in the 2D case is expected to increase to four times that of 3D case [132]. However, the dielectric constant is still that of the bulk, which in case of TMDs is rather high. The large screening reduces the binding energy to the 0.1–0.2 eV range, still allowing for a moderate degree of ionization [133]. However, if the 2D material is surrounded by vacuum, the reduced screening is expected to increase the ionization energy to the 0.5–1.0 eV range. This is comparable to the experimentally extracted exciton binding energies, a correspondence that is well known in 3D semiconductors and can be understood from the fact that both can be modelled with the hydrogenic model, albeit using different effective masses. In fact, it was recently suggested that many such defects would even “break” into charged defects and a self-trapped polaron [134]. To alleviate this, one could possibly use encapsulation by high-*k* oxides to increase the effective dielectric constant and thereby reduce the ionization energy.

All of this suggests that even the presumably hydrogenic dopants in 2D semiconductors will have ionization energies much higher than *kT*. Consequently, the resulting carrier concentrations will be minimal and likely be dominated by doping from other sources. This is qualitatively supported by experiments. Kozhakmetov et al. reported little change in the doping level of WSe_2_ with increasing Re doping up to 0.5%, with likely a metal–insulator transition taking place above 1% Re [135]. Vu et al. observed a linear increase of hole concentration in Nb-doped WS_2_ from 3%–8% [136]. Finally, Li et al. found V-doping to reduce the electron concentration of MoS_2_, which still remained n-type even at 10% V-doping [137]. As a result, compared to conventional 3D semiconductors, much higher concentrations of dopants are required to reach pronounced changes in the doping level and may be well within the metallization regime (from hybridization of dopant levels and band edges) rather than pure dopant ionization. Practically all experiments that probe carrier concentrations are carried out on insulating substrates, usually SiO_2_ or Al_2_O_3_, and tend to consistently show, for example, n-type doping for MoS_2_ and p-type doping for WSe_2_. It has been proposed that the native defects in the substrates are the primary cause of the native doping level or the charge neutrality level (CNL) [138–139]. This would agree with the observation that CNLs of different TMDs seem to align [139].

We note that it has been (erroneously) proposed that S vacancies could be the source of n-type doping of MoS_2_. This is in strong disagreement with all first-principles calculations, which show them to be deep acceptors. In addition, chalcogen vacancies in TMDs are readily passivated by oxygen in air [51,140].

### Can magnetic moments of individual defects be measured?

Solid-state spin defects have emerged as essential building blocks for quantum communication and sensing applications [141]. In 2D materials, numerous spin-active defects have been predicted and an increasing number have been experimentally confirmed [119,142–143] using optical spectroscopy, STM, and other techniques. STM is an ideal tool to measure magnetic moments of atomic-scale defects. Inelastic excitations of the spin state lead to additional tunneling channels, which is reflected in symmetric steps of the differential conductance in the d*I*/d*V* spectra [144]. In the absence of an external magnetic field, the excitation energies are directly related to the crystal-field split anisotropy of the defect in its atomic-scale surrounding [145]. The application of an external magnetic field leads to Zeeman splitting of the spin states, allowing also for the identification of the spin 1/2 states. To the best of our knowledge, no such experiments have been reported in the literature on TMDs so far. One challenge is the detection of the inelastic contribution. On the one hand, the small energy scale of at most a few millielectronvolts requires very low temperatures; on the other hand, the change in conductance depends on the excitation efficiency and the density of states at the Fermi level. Hence, detection of magnetic states in semiconducting TMDs seems out of reach, whereas it should be possible in metallic ones. Additionally, in metallic 2D materials, the magnetic moment of the defect may also be screened by the itinerant electrons giving rise to a Kondo resonance at the Fermi level. Such a spectroscopic fingerprint has been found on S vacancies in a monolayer of MoS_2_ on Au(111) [146]. However, in this case, the metallic Au substrate provided not only the electron bath for screening the magnetic moment, but also enabled the electron transfer to the vacancy due to the favorable alignment of the work functions [147]. Indeed, in areas where the MoS_2_ layer was decoupled from the metal by a Au vacancy island at the interface [148], the Kondo resonance was absent, and the spectroscopy indicated a charge-neutral state [146]. More recently, the use of STM has been extended to detect signals of electron spin resonance (ESR) in close resemblance to the conventional electron paramagnetic resonance (EPR).

Electron paramagnetic resonance (also referred to as ESR) has long been the prime method to characterize defect spins, but typically averages over billions to trillions of spins. Optically detected magnetic resonance (ODMR) has overcome this limitation by exploiting spin-selective non-radiative pathways, enabling optical initialization and single-spin readout in wide-bandgap materials [149]. Yet, ODMR is not universal: It requires both optically bright transitions and spin-dependent relaxation channels, restricting its applicability to a subset of defects. In recent years, electron spin resonance in a scanning tunneling microscope (ESR-STM) enabled to detect single spins on surfaces [150–151]. Since its first demonstration, ESR-STM has achieved coherent control of individual atomic spins with nanoelectronvolt spectral resolution [152], providing access to magnetic anisotropies, hyperfine couplings [153], and decoherence pathways at the atomic scale [151]. Extending this methodology to defects in 2D semiconductors is a natural next step: With appropriate heterostructure engineering, substrate decoupling, and integration of radiofrequency control, ESR-STM could resolve the spin fingerprints of individual defects in TMDs and h-BN, correlating atomic structure with spin states. Such measurements would establish direct correlations between atomic structure, local electronic environment, and spin properties, laying the groundwork for spin-based quantum technologies in van-der-Waals materials.

### Where are the limits for controlled defect engineering in terms of spatial precision, single type or size (e.g., hole size), and scalability?

Defect engineering in 2D materials is of obvious relevance for applications. For example, point defects can act as single-photon emitters or as qubits in quantum computing [4–5]. If one were able to position the defects precisely, all over a wafer-scale-sized 2D material, integrating the functionality of these defects into devices using subsequent lithographic processing steps would evidently be much easier. But it is not only for devices that it would be desirable to make one and the same defect with precise positioning in a 2D material over a large area. Regular defect strings or defect lattices would also enable one to bestow 2D materials with new properties that can be explored in the context of the fundamental science. For instance, a defect lattice creates a superpotential for electrons in the 2D material; thus, a mini-Brillouin zone and, consequently, a modified band structure with new electronic properties appears. The following three examples will illuminate the limits of controlled defect engineering. We will see that it is not each single factor, spatial precision, uniformity of defect species, and scalability, that creates the challenge, but their combination.

**Figure 8:**
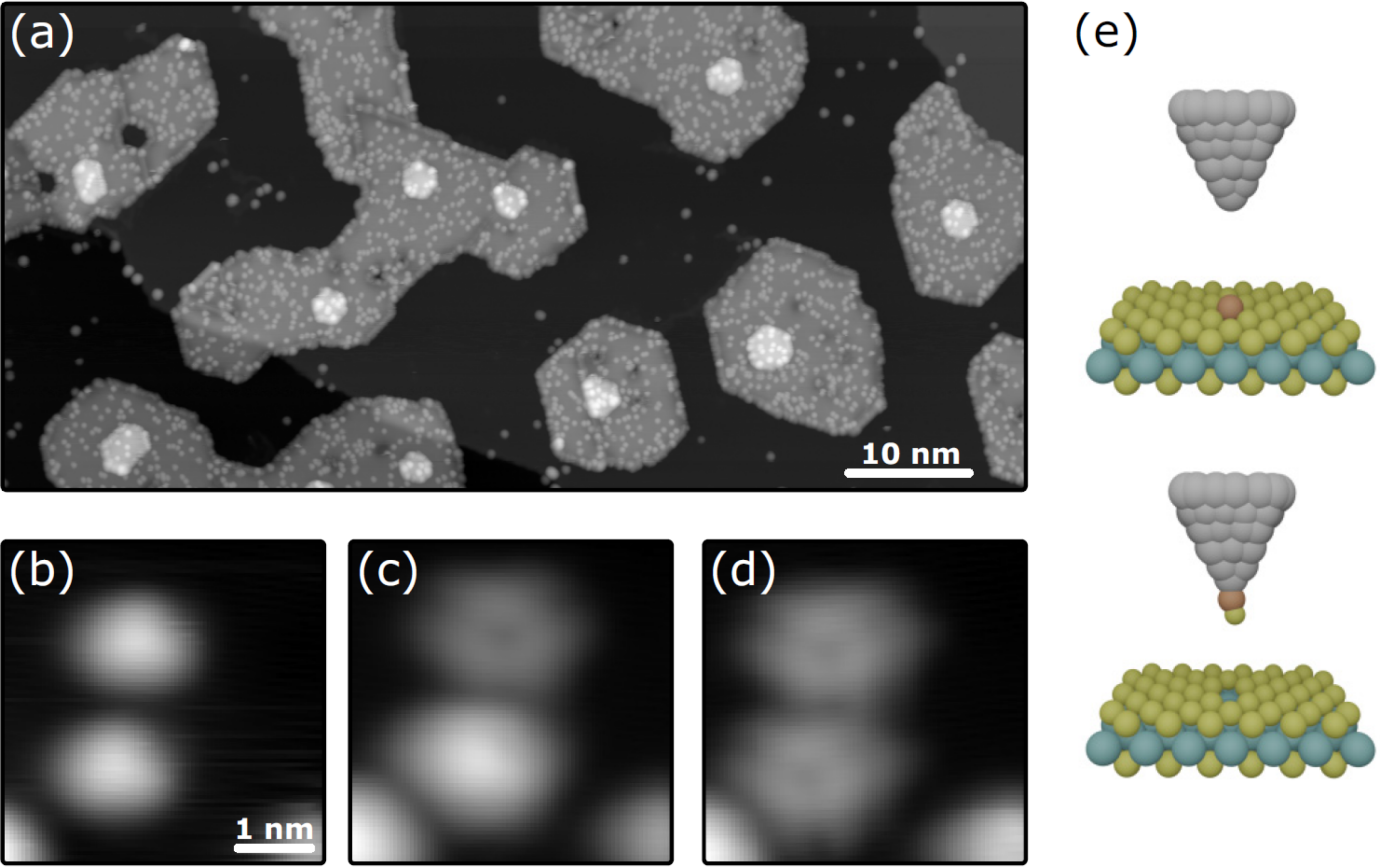
Local creation of sulfur vacancies in single-layer MoS_2_ on graphene. (a) STM topograph of MoS_2_ islands on graphene after additional deposition of a small amount of Fe atoms at 7 K. Image size: 200 nm × 90 nm. (b–d) The removal of the Fe adatoms with the STM tip is accompanied by the creation of S vacancies, whereby the removed S atom leaves the sample together with the Fe adataom. Image size: 4 nm × 4 nm. (e) Sketch of how the STM grabs the adatom together with a surface S adatom. Figure 8 was reprinted with permission from [156], Copyright 2024 by the American Physical Society. This content is not subject to CC BY 4.0.

STM is a tool that provides atomic precision and has been used successfully for atomic manipulation. Recent experiments were able to create extended lattices of CO molecules on Cu(111), whereby, for instance, the surface-state free electron gas could be transformed into an electronic structure mimicking the one of graphene [154]. Also, precise manipulation at large scale (thousands of defects) for vacancies in a chemisorption layer of Cl on Cu(111) was reported [155]. These experiments suggest that it could be possible to apply such atomic manipulation methods to 2D materials. Figure 8 provides an example of how this can be accomplished. Figure 8a is an STM topograph of MBE-grown MoS_2_ islands on graphene, on which, after growth and at about 7 K, a small number of additional Fe atoms were deposited. On MoS_2_, these atoms are immobile after deposition. Approaching an Fe adatom with the STM tip until a sudden change in the measured tip height is detected removes the Fe adatom from the MoS_2_ and transfers it to the STM tip. When leaving the sample surface, each Fe adatoms rips out an S atom of MoS_2_ such that a single S vacancy is left behind. The method has a very high probability of success and creates one and the same vacancy defect. If the adatom species could be easily moved laterally with the STM tip on the 2D material (unlike Fe adatoms on MoS_2_), one could create strings and lattices of single vacancy defects with atomic precision, thereby effectively making a material with new global properties. It is likely that a suitable 2D material–adatom combination exists. One even might speculate that, using a properly functionalized tip, the last tip atom itself could be used to create the vacancy. Speculating further, using STM tip manipulation methods, it appears possible not only to create single vacancies but also to fill a vacancy with a foreign atom, a defect that may be almost impossible to create otherwise. In the next years, such investigations are likely to happen, and we can expect to gain deep insight into the physics of the created defects and defect arrays using high-resolution STS. However, it is obvious that the method lacks scalability. At least today, it is hard to envision how using an STM tip a useful defect concentration could be induced into wafer-scale substrates.

**Figure 9:**
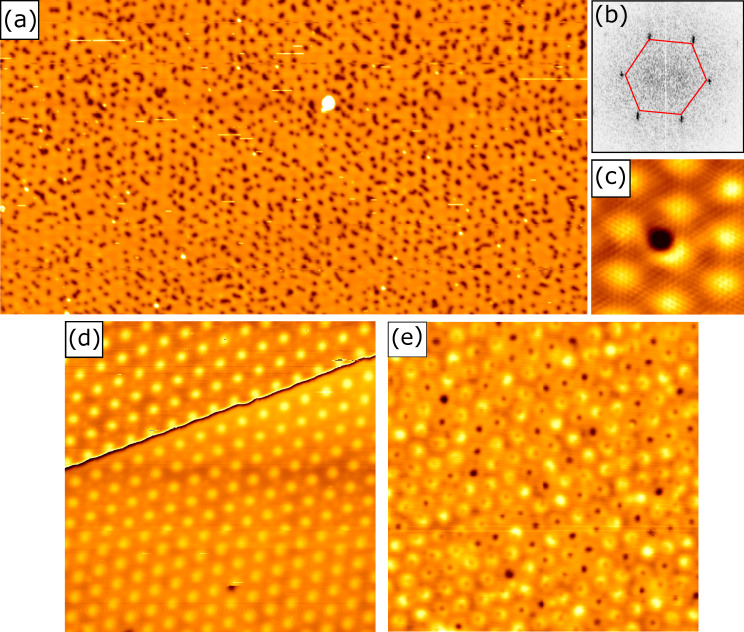
Self-organization of vacancies created by ion irradiation of single-layer graphene (Gr) or hexagonal boron nitride (h-BN) on Ir(111). (a) Gr/Ir(111) after exposure to a He^+^ fluence of 1.2 × 10^19^ ions/m^2^ at 1150 K. Imaged by STM at 300 K. Image size 110 nm × 60 nm. (b) Fast Fourier transform of (a) with peaks corresponding to the hexagonal array of vacancy clusters with a periodicity of 2.5 nm defined by the Gr moiré. (c) Atomic-resolution topograph of the Gr/Ir(111) moiré with a single vacancy cluster. Image size 7 nm × 7 nm. (d) STM togograph of single-layer epitaxial h-BN on Ir(111) prior to ion irradiation. The hexagonal moiré lattice with a periodicity of 2.9 nm is well visible. (e) Sample as in (d) after an additional 500 eV He^+^ ion fluence of 1.2 × 10^19^ ions/m^2^ at 1200 K. Imaged by STM at 300 K. Image size for (d) and (e) is 45 nm × 45 nm. Figure 9d–e was reprinted with permission from [157], Copyright 2022 by the American Physical Society. This content is not subject to CC BY 4.0.

Scaling is inherently possible using self-organization. The next example provides an impressive combination of spatial order, size uniformity, and scalability in a 2D material based on self-organization. When single-crystalline graphene (Gr) on Ir(111) is irradiated with noble gas ions of sufficiently low energy, such that only single Gr vacancies are created, and at sufficiently high temperature, such that the vacancies are mobile, the vacancies organize into clusters of rather uniform size, whose positioning reflects the moiré pattern of Gr with Ir(111) as shown in Figure 9a [157]. The fast Fourier transform of the topograph in Figure 9b confirms the visual impression from Figure 9a. It displays clear “diffraction” peaks at the locations corresponding to the moiré lattice. The formation of a partially ordered nanomesh with size-limited pores is based on the finding that vacancy clusters are unable to bind with their dangling bonds down to the substrate in a specific region of the moiré unit cell (top region) [158]. This region is bright in the STM topograph of Figure 9c. Thereby, these locations are energetically unfavorable to host vacancy clusters. The clusters are also size-limited, as clusters growing too large and into the bright areas would force Gr bonds to detach from the substrate. Therefore, vacancies diffusing around avoid attachment to large clusters and prefer to attach to smaller ones, making the size distribution rather uniform. The average vacancy cluster size in Figure 9a is twelve vacancies. A similar mechanism of vacancy cluster self-organization applies to the moiré of h-BN with Ir(111) [157]. Figure 9d displays the pristine h-BN moiré with pronounced bright spots. Upon 500 eV He^+^ ion irradiation at 1200 K, vacancy clusters (dark spots of varying size) are found to be exclusively attached to the bright areas of the moiré. They form indeed a lattice with about 90% of the sites occupied. Transfer of such nanomeshes to a foreign substrate [157] makes it possible to explore their use in membrane applications like water desalination or gas purification.

**Figure 10:**
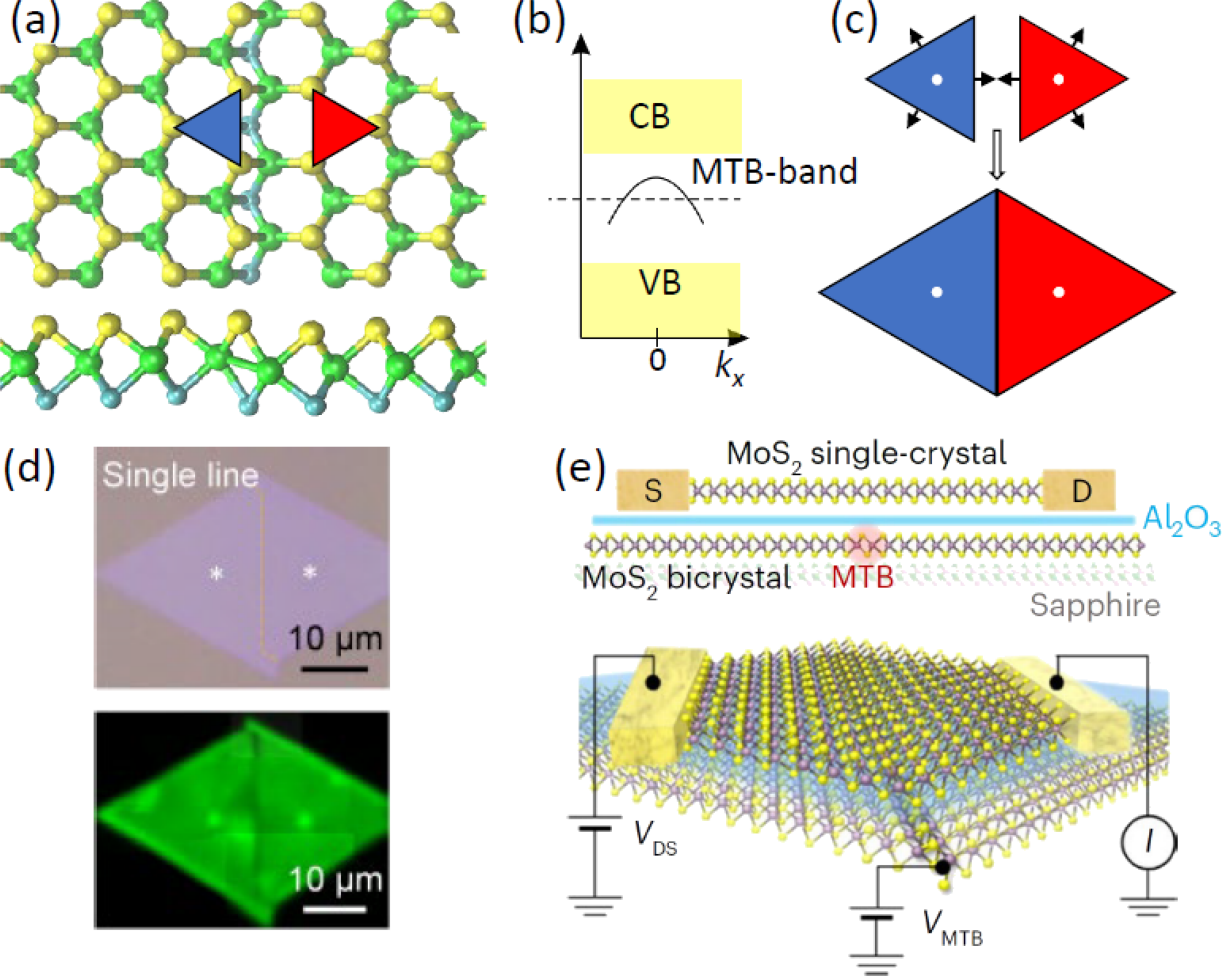
Mirror twin boundaries (MTBs) as gate electrodes in field-effect transistors (FETs). (a) Top- and side-view atomic ball model of a mirror twin boundary in single-layer MoS_2_. Red and blue triangles highlight the mirror symmetry. (b) Schematic sketch of the hole-like band dispersing along the MTB located in the bandgap between valence band (VB) and conduction band (CB). (c) Growth of mirrored triangular MoS_2_ islands away from the prefined nucleation sites (white dots) leads to a twin boundary. It is located halfway on the line connecting the nucleation sites and normal to it. (d) Upper panel: optical micrograph of two mirrored triangular MoS_2_ islands grown together forming a MTB. The position of the MTB is indicated by the dashed yellow line. Lower panel: MTB made visible as dark line by second harmonic generation imaging. (e) Side- and perspective-view sketch of a field-effect transistor using a MTB as gate electrode. S: source; D: drain; *V*_DS_: drain–source voltage; *V*_MTB_: gate voltage applied to the MTB; I: drain–source current. Figure 10 was adapted with permission from Springer Nature from [164], by W. Jolie et al., “1D metals for 2D electronics”, *Nature Nanotechnology*, Vol. 19, Pages 883–884, 2024. Published by Springer Nature. This content is not subject to CC BY 4.0.

The last example illuminates how a more complex 1D defect in a 2D material can be created with good spacial precision and scalability, making the defect suitable for application. When two 2D crystals mirrored along a line are joined, the twin crystal contains a mirror twin boundary as a 1D line defect, in full analogy to a 3D twin crystal with a 2D twin plane. An atomic model of a mirror twin crystal with a MTB as formed in single layers of MoS_2_ is shown in Figure 10a. Such MTBs emerge when the threefold-symmetric MoS_2_ is grown on a sixfold-symmetric substrate [159–160]. While the MTBs possess many exciting properties of fundamental interest [159,161–163], in the present context, it is only important that translational symmetry along the line defect is preserved, giving rise to a dispersing hole-like band as shown in Figure 10b, that is, to a 1D conductor embedded into a 2D semiconductor. To make use of such ultimately thin conductors, it is necessary to define their position. In the epitaxy of MoS_2_ on *c*-plane sapphire, this is accomplished by predefinition of nucleation sites using a focused ion beam (white spots in the schematic sketch of Figure 10c [160]). After nucleation of two mirrored islands and upon further growth, a MTB forms halfway between the triangular MoS_2_ islands. An example of such predefined MTB formation is shown in Figure 10d. This close to deterministic MTB positioning enabled their use as ultrashort gate electrodes in arrays of field-effect transistors (FETs), of which one is schematically sketched in Figure 10e [160,164]. The beauty of the use of MTBs in 2D FETs results from the fact that the construction of the FETs is much simpler than in previous ultrashort-gate FETs [165–166] since the gate electrode, the MTB, is already formed and positioned during growth, with at least equal performance. Preconditions for making use of the MTBs in FETs are the defined spatial positioning of the MTBs together with their inherent uniformity and perfection. The three examples highlight that we are indeed about to reach control over defect positioning, uniformity (only one and the same defect present), and scalability, which ultimately will lead to useful technological applications.

### What are the limitations of X-ray photoelectron spectroscopy in identifying and quantifying defect concentrations in 2D materials, and how can these limitations be addressed?

X-ray photoelectron spectroscopy (XPS) is a powerful tool for identifying and quantifying defects in 2D materials, offering unique advantages in surface sensitivity, chemical state analysis, and real-time monitoring of defect evolution. While challenges such as surface contamination, depth sensitivity, energy resolution, and data complexity exist, they can be addressed through careful sample preparation, the use of complementary techniques, and the integration of theoretical modelling for data analysis. The ability of XPS to study defect evolution under specific atmospheric conditions further broadens its applicability, making it an invaluable tool for understanding and optimizing the properties of 2D materials. Continued advancements in XPS technology, including integration with in situ/operando capabilities, microscopy, and machine learning for data analysis, promise to further enhance defect characterization and accelerate the development of high-performance materials for various applications.

The fundamental principle of XPS lies in its ability to probe the binding energies of core electrons, which are influenced by the local electronic environment surrounding the atoms [167–171]. Essentially, by analyzing shifts in binding energies and changes in peak intensities, XPS can identify various defect types, including vacancies, interstitials, and substitutional defects [172–178]. In the context of 2D materials, defects exhibit distinct signatures in the XPS spectra, enabling their identification. By meticulously analyzing the XPS spectra in conjunction with DFT analysis, researchers can determine the nature of defects present in the material, distinguishing between different types based on the observed binding energy shifts and peak shapes [179–187].

Complementing XPS, scanning X-ray photoelectron microscopy (SPEM) provides spatially resolved chemical information at the nanoscale, and near-ambient pressure XPS enables real-time monitoring of the chemical reactivity of defects in 2D materials under various environmental conditions [187–192]. Despite its strengths, XPS has several limitations that must be addressed for accurate defect characterization. One major challenge is surface contamination, which can obscure defect signals. Hydrocarbon residues and other contaminants may alter the XPS spectra, leading to misinterpretation of defect characteristics. To mitigate this, careful sample preparation in controlled environments and in situ cleaning methods, such as argon ion sputtering, are essential. However, these approaches must be applied cautiously to avoid introducing new defects or modifying existing ones. Another limitation is the depth sensitivity of XPS. The electron escape depth (typically 1–10 nm) is larger than the thickness of most 2D materials (0.5 to 0.8 nm for a monolayer); therefore, XPS may include contributions from underlying substrates or adjacent layers in multilayered structures. Techniques such as angle-resolved XPS may address this issue by varying the photoelectron emission angle, enabling depth profiling and providing insights into defect distributions across different layers [193–195].

**Figure 11:**
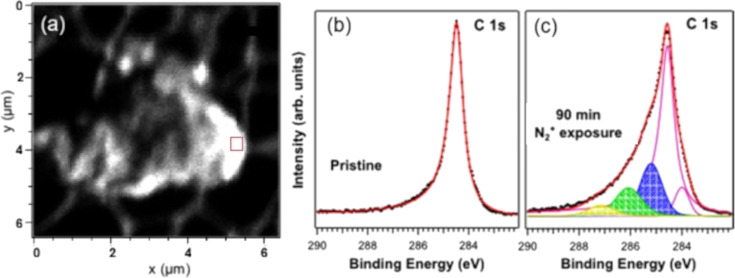
(a) 6.4 × 6.4 mm^2^ scanning photoemission spectroscopy (SPEM) image of a graphene flake recorded at the C 1s energy. The XPS spectra were recorded in the region represented by a red square. Spatial resolution 100 nm. C 1s spectrum recorded (b) before and (c) after nitrogen ion irradiation for 15 min. Black dots represent the experimental data, and the red continuous line is the result of the least-squares fit procedure. Figure 11 was adapted from [188], *Carbon*, Vol. 73, by M. Scardamaglia, B. Aleman, M. Amati, C. Ewels, P. Pochet, N. Reckinger, J.-F. Colomer, T. Skaltsas, N. Tagmatarchis, R. Snyders, L. Gregoratti, C. Bittencourt, “Nitrogen implantation of suspended graphene flakes: Annealing effects and selectivity of sp2 nitrogen species”, Pages 371–381, Copyright (2014), with permission from Elsevier. This content is not subject to CC BY 4.0.

The complexity of defect types in 2D materials also poses challenges for interpreting the data. Overlapping signals from different defects, such as vacancies, interstitials, and substitutional defects, can complicate analysis. To address this, combining XPS with complementary techniques, such as Raman spectroscopy, AFM, and DFT analysis, can provide a more comprehensive understanding of defect characteristics. Quantitative defect analysis is further hindered by the variability in XPS signal intensity, which can be influenced by factors such as surface roughness and electron escape depth. Calibration against standard samples with known defect concentrations can improve accuracy. For instance, Scardamaglia et al. reported on defect characterization in nitrogen-implanted graphene through a detailed analysis of XPS data recorded on nitrogen-ion-irradiated suspended graphene, specifically focusing on the C 1s core-level spectra [174,188]. The presence of defects, including vacancies and sp^3^-hybridized carbon, was inferred from the observed broadening and shifting of the C 1s peak. As shown in Figure 11, in pristine graphene, the C 1s peak appears as a sharp, well-defined feature. However, in the nitrogen-doped samples, the C 1s peak exhibits significant broadening, indicating structural disorder within the graphene lattice [188]. Five components were used to reproduce the C 1s spectrum after the nitrogen irradiation: The asymmetric component, peaking at 284.47 eV, was reported to be representative of carbon atoms in sp^2^ C–C bonds in pristine suspended graphene. The component with the lowest binding energy at 283.70 eV was associated with vacancies in the carbon lattice, while the one at 284.80 eV is attributed to sp^3^ carbon hybridization. The other two components are associated with C–N bonds in different configurations.

**Figure 12:**
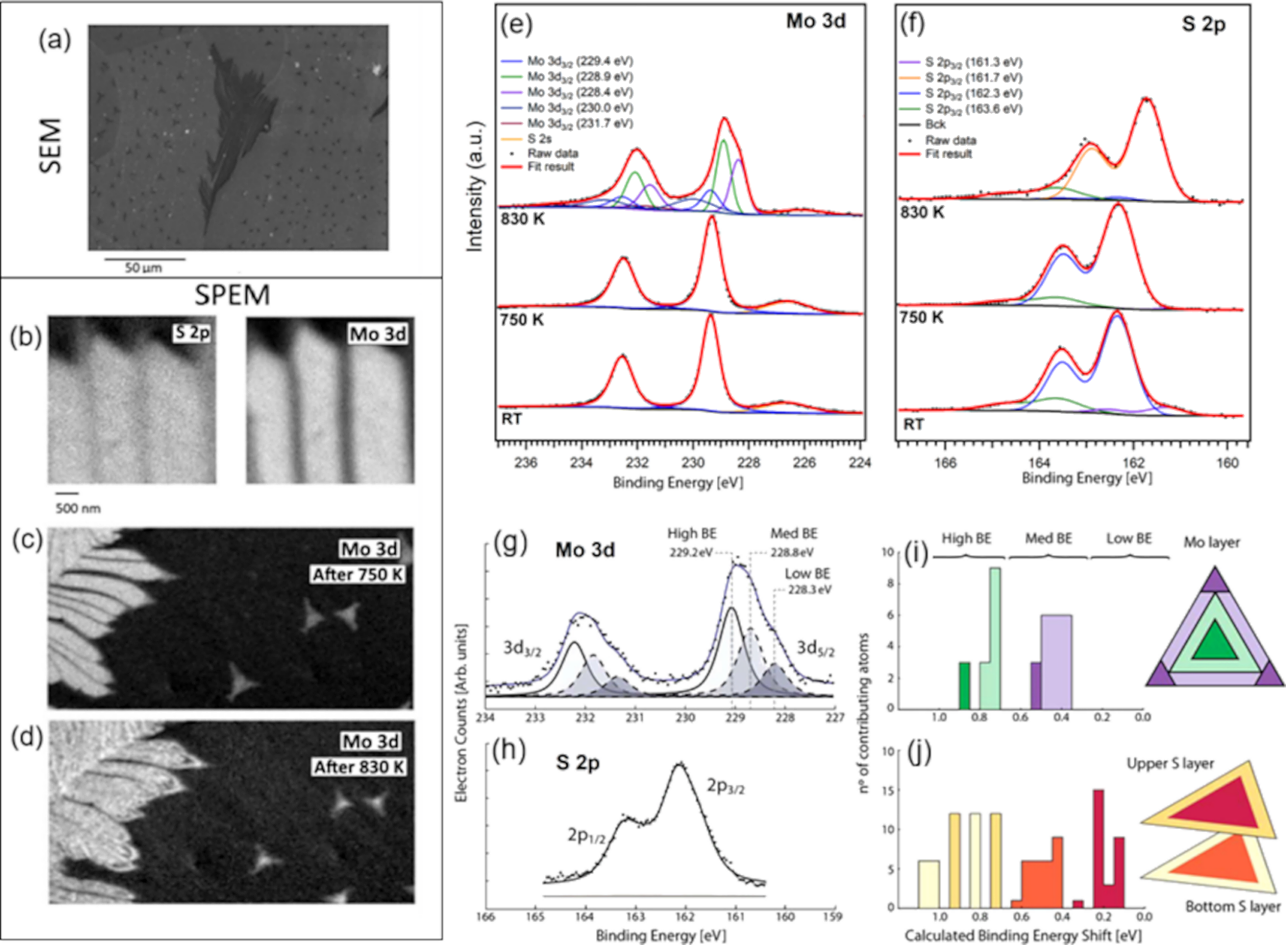
(a) SEM image of a MoS_2_ film grown on Au foil. (b) SPEM Mo 3d and S 2p maps of the pristine sample. Mo 3d map after annealing at (c) 750 K and (d) 830 K in H_2_. XPS spectra for (e) Mo 3d and (f) S 2p of MoS_2_ fractals at room temperature (RT), as well as during annealing at 750 and 830 K in a 1 mbar H_2_ atmosphere. Deconvolution of XPS spectra and DFT-calculated binding energy shifts: (g) Mo 3d and (h) S 2p XPS spectra, alongside DFT-calculated core-level binding energy (BE) shifts for MoS_2_ nanoparticles supported on Au(111), (i) for Mo 3d and (j) for S 2p. The fitted Mo 3d spectrum is separated into three distinct components (high, medium, and low BE), which are correlated with the calculated shifts. The various colors in the histograms represent the positions of Mo and S atoms (corner, edge, near-edge, or basal plane) within the MoS_2_ triangle, indicating their contributions to the observed Mo 3d and S 2p shifts, as in the corresponding triangle diagrams. Figure 12 panels (a–f) were reproduced from [186] (© 2023 M. Amati et al., published by Elsevier B.V., distributed under the terms of the Creative Commons Attribution 4.0 International License, https://creativecommons.org/licenses/by/4.0). Figure 12g–j was reprinted with permission from [201]. Copyright 2015 American Chemical Society. This content is not subject to CC BY 4.0.

The integration of XPS data with theoretical calculations, such as DFT, further enhances the interpretative power of the technique, enabling a robust identification of defect types and their associated chemical states [179–187]. For instance, Amati et al. used SPEM to evaluate defect formation in 2D MoS_2_[186]. The poor catalytic activity of TMCs can be enhanced by introducing intrinsic or extrinsic defects, including vacancies and dopants [132,196–198]. In this context, SPEM was applied to in situ investigate a monolayer of MoS_2_ grown on a gold foil during thermal annealing in hydrogen, reported as a method for creating sulfur vacancies [186,199]. The pristine Mo 3d_5_*_/_*_2_ spectrum was reported to display a single component at 229.4 eV, typical of stoichiometric 2H-MoS_2_. As the temperature increased, significant changes were observed in the XPS spectra, particularly at 830 K, where new XPS spectral features emerged. Two major components were reported to appear in the Mo 3d spectrum at binding energies of 228.9 and 228.4 eV, associated with the presence of undercoordinated Mo species associated with sulfur vacancies [186,200]. The corresponding S 2p spectrum showed a primary peak at 162.3 eV, indicative of sulfide species, along with additional peaks at 161.3 eV, attributed to elemental sulfur on the gold support, and at 163.6 eV, which can be linked to SO*_x_* species. The SPEM image shows that the reaction with hydrogen initially occurred at the edges of the islands, leading to the formation of vacancy islands as sulfur atoms were eliminated. In another work, Bruix et al. [201] examined the edges of in situ grown MoS_2_ islands on Au(111), revealing how different atomic contributions affect the spectral characteristics of the Mo 3d and S 2p core levels (Figure 12, [186,199]). By combining XPS with DFT calculations, they identified multiple shifts in the Mo 3d_5_*_/_*_2_ core level, corresponding to Mo atoms at various positions within the triangular MoS_2_ islands. The interaction with the Au substrate caused the S 2p doublet to split into two peaks, namely, one at higher binding energy for sulfur atoms in contact with Au, indicating S–Au bond formation, and another at lower binding energy from the upper sulfur layer. Monitoring the XPS signal during hydrogen exposure provided insights into the reduction process decreasing sulfur coverage, which resulted in a shift of the Mo 3d core level to higher binding energy, attributed to changes in the metallic character of Mo.

### Do the calculated and experimentally measured concentrations of irradiation-induced defects agree?

**Figure 13:**
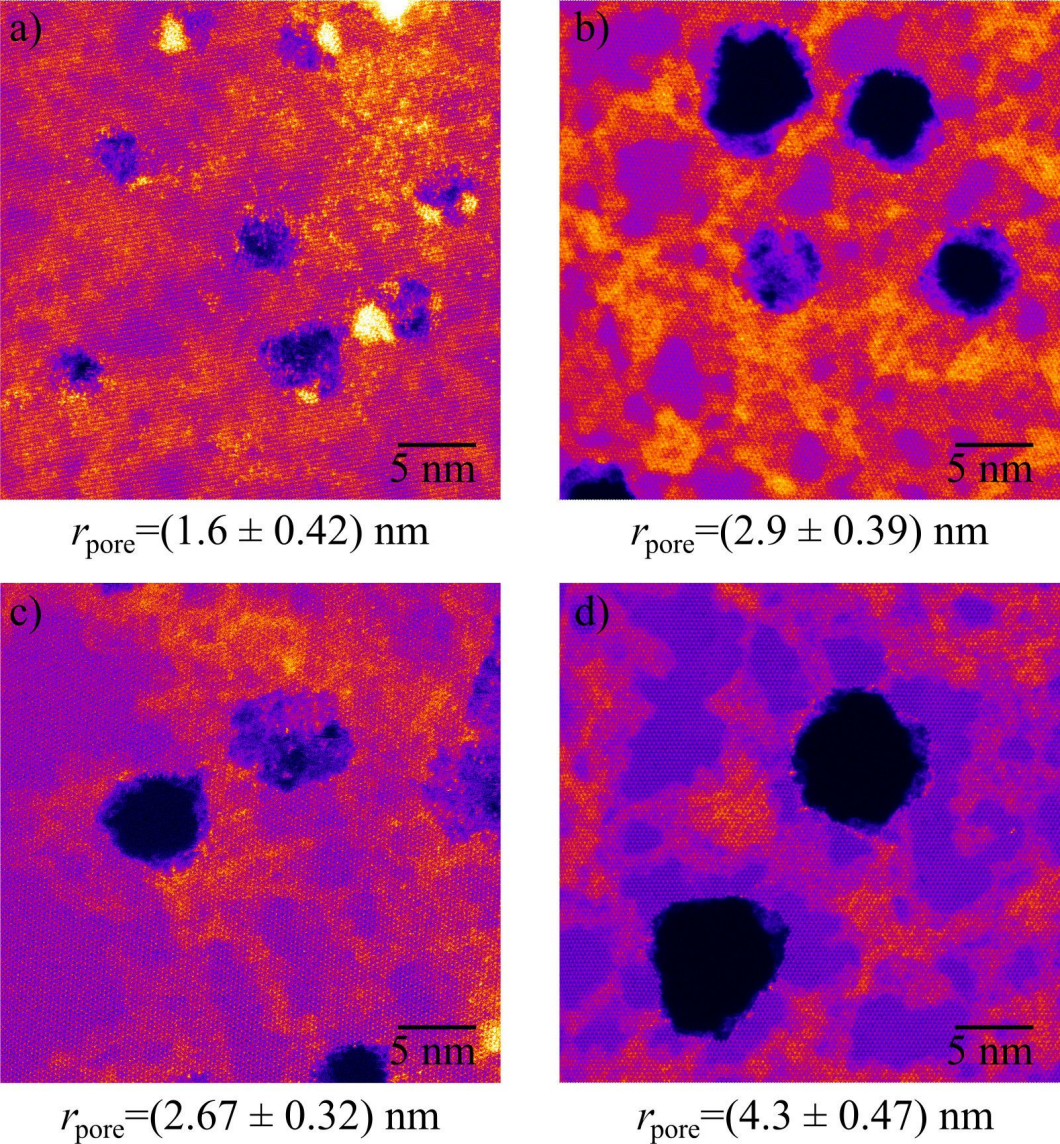
Atomically resolved STEM images of ion-induced pores in MoS_2_ with dominant electronic excitation. (a, c) suspended and (b, d) supported on SiO_2_. Supported monolayers have been transferred onto TEM grids after irradiation. (a, b) 0.7 MeV/u Xe^23+^ and (c, d) 180 kV Xe^40+^. Images courtesy of Yossarian Liebsch (AG Schleberger), University of Duisburg-Essen and Umair Javed (AG Kotakoski), University of Vienna.

Ion irradiation is a highly versatile tool for defect engineering in 2D materials since the modifications depend sensitively on ion type, energy, and charge state. Energy deposition occurs either via direct collisions (nuclear stopping) or through electronic excitations (electronic stopping). For simplicity, three scenarios are typically distinguished: (I) slow, singly charged ions (e.g., Ar^+^ with kinetic energies in the range of 50–5000 eV), (II) slow, highly charged ions (e.g., Xe^40+^ in the range of 50–500 keV), and (III) fast (swift) heavy ions (e.g., Xe^23+^ in the range of 100–1000 MeV). All three regimes have been used to introduce defects in 2D materials [7]. Scenario I mainly produces small defects such as chalcogen vacancies via atomic collisions, whereas scenarios II and III enable the formation of extended defects, that is, pores with radii ranging from a few angstroms to several nanometers, depending on the irradiation parameters. Figure 13 shows atomically resolved examples of such ion-induced defects in MoS_2_. Quantifying the nature and size of ion-induced defects is experimentally challenging as most irradiation experiments are performed on 2D materials supported on substrates. This prevents the use of high-resolution techniques such as STEM. Alternatives like scanning probe microscopy suffer either from substrate limitations (STM and AFM) or insufficient resolution under ambient conditions (AFM). Substrate effects further complicate the picture. Recent STEM studies of supported MoS_2_ revealed slightly larger pores, as can be seen in Figure 13. In principle, this is consistent with the theoretical predictions and attributable to backscattering processes; however, for a quantitative comparison, experimental data with atomic resolution, in particular after scenario-I irradiation, is still too scarce.

As for using high-resolution TEM for defect concentration assessment, the concentration of defects can be probed locally, giving an indication of the overall material. Considering impurities, their number in the material after irradiation will give the retention rate, that is, how likely their formation upon irradiation is. The rate highly depends on the irradiation parameters such as ion energy. The local variation in the level of contamination present on the 2D surface also has a significant effect on the experimental retention rates. The ion may end up within or under the contamination, lowering the number embedded in the 2D material itself. Local variations in the retention may also rise from the slow moving particles as ultralow energies are required for the implantation into 2D membranes. Such slowly moving projectiles could be deflected, for example, if the contamination becomes charged. These effects are not considered in the theoretical predictions. Nevertheless, experiments with B- and N-implanted freestanding graphene exhibit good agreement with theoretical predictions. The overall retention rate for 200 eV N range from 5% to 15% of the implanted dose [11]. This compares well to the theoretical prediction of 5% [202]. Experimentally 100 eV B-implanted clean regions show retention of the order of 15% [11], and theory matches with 15% using DFT [203]. When areas of graphene with contamination are included, the experimental retention goes up to 50%. Lower implantation energies (25 eV) can achieve as high as 100% retention rate for N; however, the number goes down to 15% or even lower in clean areas (local concentrations as low as 0.5% were recorded). The calculated values match the estimate for clean graphene, indicating 15% retention [203]. For Se substitution into the S site of a freestanding MoS_2_ monolayer, a retention rate of 1% has been reported with 10 eV ion energy [23]. Calculations, however, indicate a retention rate for substitutional sites at about 9%, which goes as high as 40% when the ion energy is increased to 25 eV [15]. The discrepancy between theory and experiment becomes larger as the systems become more complex when a substrate is included. Supported graphene implanted with 25–100 eV N and studied using electrical testing [204] indicate an increase in electron field-effect mobility attributed to substitutional nitrogen doping, indicating a retention rate of about 3%. Using a much heavier projectile, a retention rate of 1% (Mn 40 eV) [12] and 10% [16] (Mn 60 eV) in supported graphene have been recorded experimentally. For comparison, ab initio MD indicates retention of about 45% [16], which depends strongly on the moiré supercell at the point of the impact. Differences in timescales and subsequent experimental processing like annealing, are likely contributing factors in the variation between theoretical and experimental results.

For introducing dopants that have a large mass difference between the ion and the target, a two step process is used due to inefficient momentum transfer in a one step “direct” implantation [21]. The method relies on first creating vacancies by ion irradiation then filling them by landing the desired impurity atoms onto the vacancy-ridden 2D material. The total retention rate is a combination of the efficiency to create vacancies and the efficiency to fill these. For free-standing graphene with gold dopants, it is reported to be 0.2% in the area of clean graphene [20].

### Do we fully understand the mechanism of the neutralization of highly charged ions after passing through 2D materials? How does the neutralization depend on the electronic structure of the system?

A dynamic change of the ion charge state due to the interaction with a solid surface is governed by a complex interplay of charge transport between the ion and the surface [205], charge density rearrangement in the surface area [206–207], and Auger-type de-excitation processes [208–209]. While swift heavy ions typically increase their charge by friction with the electronic system of a solid, it is not yet clarified how this charge exchange affects the electronic stopping power responsible for swift heavy ion-induced surface modifications and perforation in 2D materials (see Figure 13a,b).

For slow ions, that is, if the ion velocity is well below the electron Fermi velocity, the ionic charge is neutralized very efficiently within one to three monolayers of graphene [210]. It has been shown that the neutralization follows an exponential charge decay over time (i.e., it depends only implicitly on the ion velocity). The ions show the same charge exchange in single-layer graphene as in bilayer graphene at twice their velocity. This finding also implies that the target atomic lattice, that is, the number of atoms the ion passes ultimately defines the neutralization. An atom-centered interatomic Auger decay, like interatomic Coulombic decay [211–212] of a highly excited atom/ion can, in principle, explain the empirical findings of charge neutralization of heavy highly charged Xe ions in graphene [213]. For highly asymmetric ion–target atom combinations, like Xe on C, a substantial level mixing via molecular orbital formation is not expected, and interatomic Auger decay remains the dominant mechanism to transfer the ion potential energy to the surface. It further stabilizes captured electrons through de-excitation almost to the ground state of the ion. For other, less asymmetric cases, molecular orbital formation cannot be ruled out and might become significant [214–215]. A quasi-molecule formation during the collision of a slow ion with a target atom would lead to a different time dependence of charge exchange and should show a clear velocity threshold for these charge exchange channels to open. A different mechanism for charge exchange could furthermore imply a different efficiency for potential energy deposition and, consequently, affect pore formation in TMDs.

**Figure 14:**
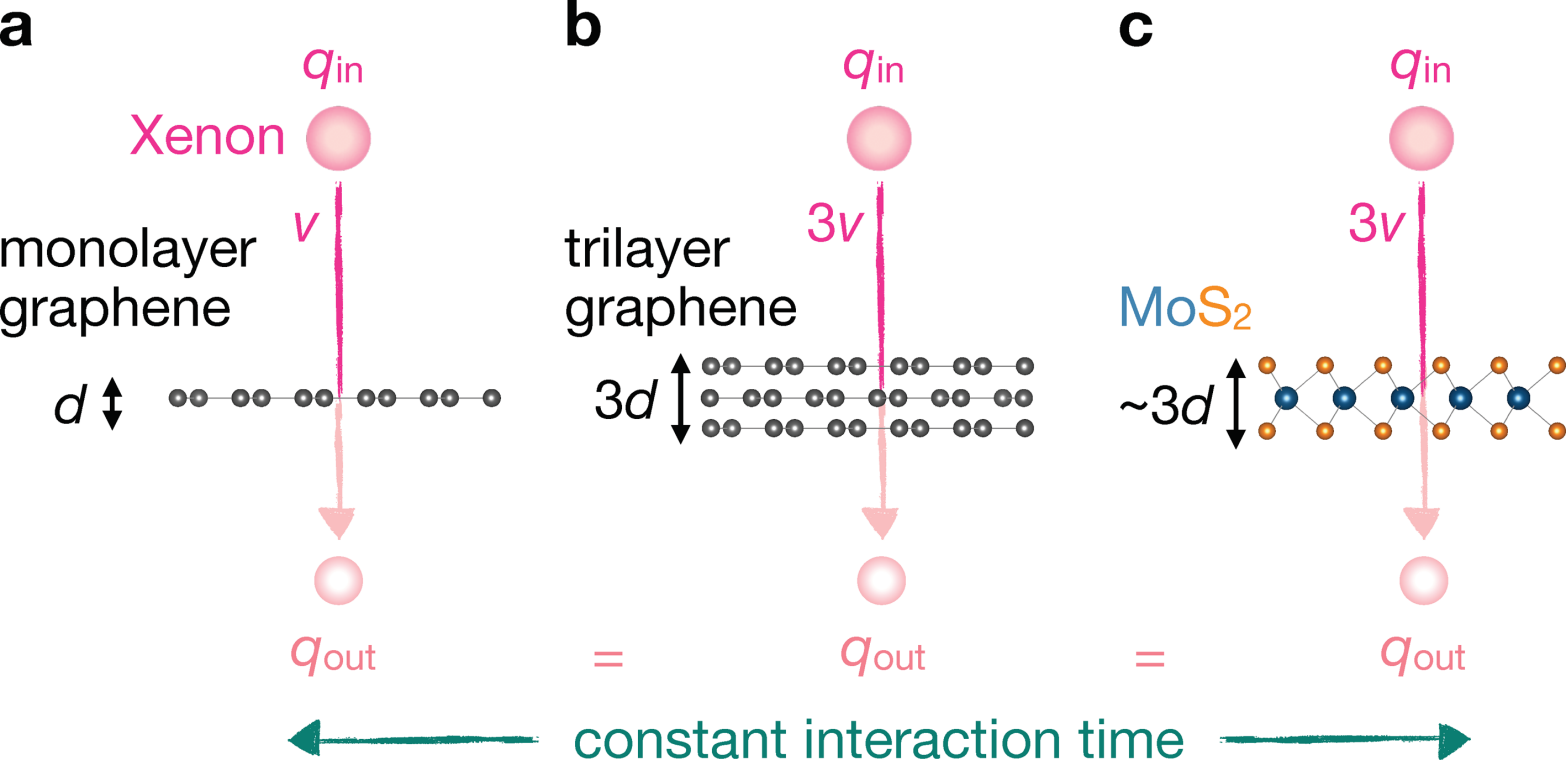
Schematic of the charge exchange of highly charged (xenon) ions through 2D materials. The charge exchange is governed by the interaction time of the ion with the material layer(s): For the same incident charge state *q*_in_, the same outgoing charge state *q*_out_ is reached when (a) the ion has velocity *v* and transmits a monolayer of graphene, (b) the ion has velocity 3*v* and transmits a trilayer of graphene, and (c) the ion has velocity 3*v* and transmits a monolayer of MoS_2_. In all three cases, the interaction time *t* = *d*/*v* with the material thickness *d* is similar. Material layers were rendered using the software Vesta [218].

Previous studies of highly charged Xe ion transmission through graphene and freestanding MoS_2_, however, showed that MoS_2_ provides similar neutralization as graphene for Xe ions [216], considering the increased number of atomic planes. The three-atomic-layer-structure of MoS_2_ shows similar charge exchange as a trilayer graphene membrane (Figure 14). Furthermore, the charge-exchange-enhanced ion stopping power [217] is also similar between graphene and MoS_2_ if scaled to the same amount of atomic planes (three in the case of a monolayer of MoS_2_ and one in the case of graphene). We can therefore infer that it is in fact the number of atoms the ion passes that determines the amount of charge exchange and of deposited potential energy. There is, however, a lack of experimental data for other 2D materials and other ionic species with some preliminary data (unpublished) for Ar ions and h-BN monolayers. For Ar ions, the ionization of the L and K shells is accessible in experiments, while for slow Xe ions, typically, the L and K shells cannot be ionized. A jump in ionization potential at Ar^8+^ and Ar^17+^ reflects in a different exponential decay (neutralization time constant) in graphene. While this data demands further clarifications, it indicates a strong influence of the atomic level structure and occupation of the ion on the charge exchange. When the target system is modified, that is, a wide-bandgap insulator like h-BN is used, no significant differences in the charge exchange for Xe ions were observed in comparison to graphene.

The similarities in the charge exchange between graphene and MoS_2_ (and h-BN based on preliminary data) suggest that the band structure of the target material is not a determining factor for the ion neutralization and potential energy deposition. The perforation of 2D materials or modification of the surfaces, however, depends very strongly on the electronic structure of the solid. For lattice modifications, two steps should be considered, namely (i) the ion neutralization and potential energy deposition, which might be largely independent of the specific surface material, and (ii) the surface response to the strong electronic excitations, which appears largely different for (semi)metals, semiconductors, and insulators. It should be noted, however, that a comparison between different materials always includes different band structures, different atomic species, different lattice structures, different atomic binding energies, and different types of charge hopping. A fair comparison between metallic and insulating surfaces to attribute differences in perforation to the material and its electronic structure should involve surfaces with a metal-to-insulator transition, keeping material composition and lattice structure largely unchanged.

### Can holes with controllable sizes be produced using energetic ions?

Creating “perforated” 2D materials with a narrow distribution of hole diameters is highly desirable for various applications like DNA sequencing, molecule sieving, or water purification [219–224], but manufacturing such materials is challenging. This problem can be circumvented by impacting freestanding 2D materials, typically prepared on TEM grids, with energetic ions. Pores of the order of a few nm in radius created in such freestanding samples by electronic excitation (scenarios II and III) are usually circular with narrow size distributions. For freestanding MoS_2_, irradiation with highly charged ions resulted in a linear increase in pore size with potential energy depending on the ion’s charge state [225]. In the case of swift heavy ions, the pore size as a function of energy deposition per layer, controlled by the ion kinetic energy, follows a power law [226]. The latter observation is noteworthy for two reasons: (i) Such power-law behavior is expected if electronic excitations couple to phonons, as described by the two-temperature model; (ii) it is obscured when the stopping power is estimated using SRIM [227]. While SRIM is widely used to evaluate electronic stopping, it is based on semi-empirical fits and neglects relevant contributions such as electron bunching, spatial straggling, and energy carried away by escaping particles. Although a more accurate model has been conceived by Liebsch et al. [226], the exact description of the initial energy deposition via electronic excitation remains unresolved. Another issue is the efficiency of pore creation. Typically, the number of pores per incident ion is below unity. Near-unity efficiency can be achieved, for example, for highly charged ions at very high charge states [225] or for swift heavy ions with sufficiently high electronic stopping [226].

### Are 2D materials radiation-tolerant, as discussed in the literature in the context of transistor operation?

**Figure 15:**
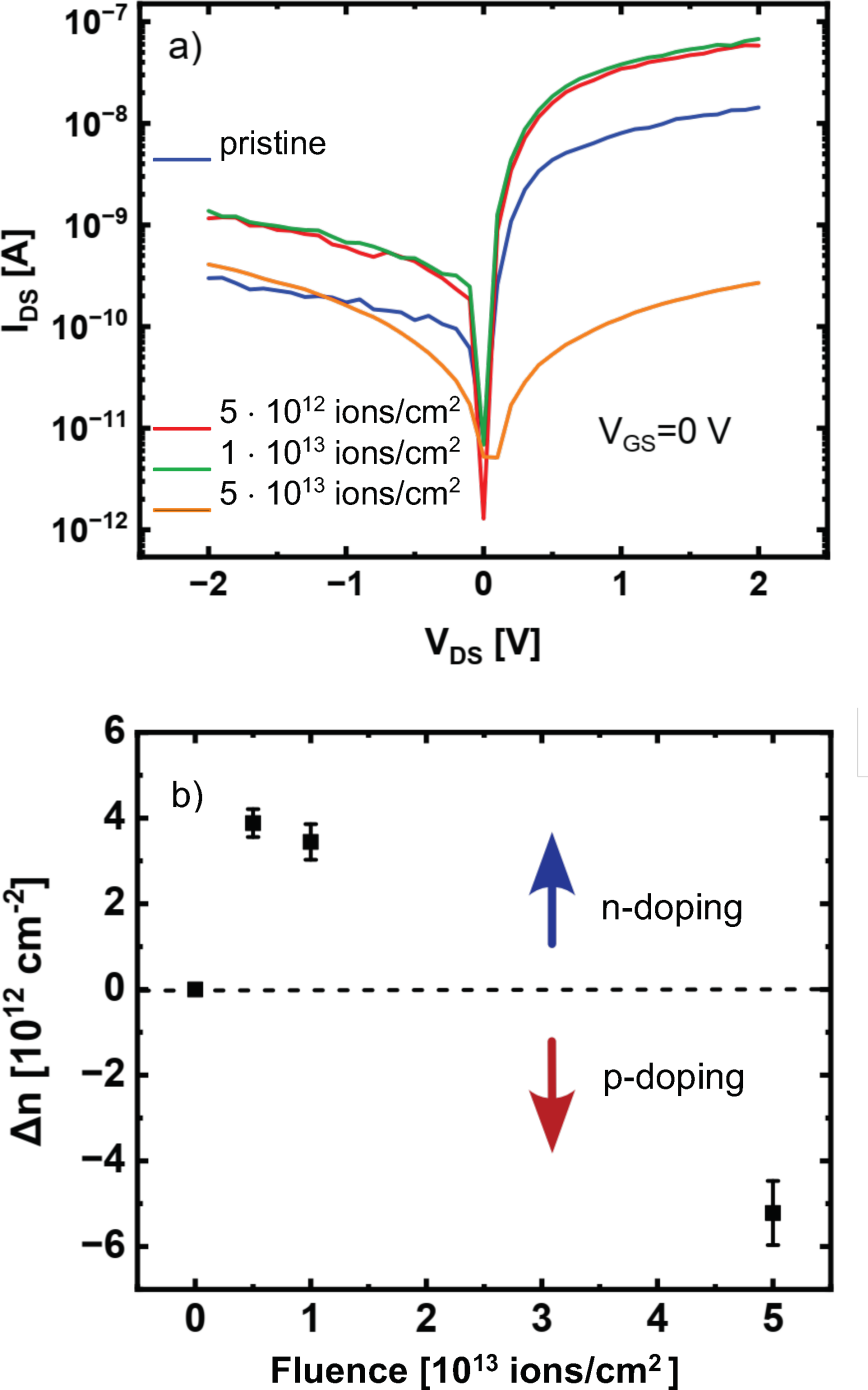
(a) Output characteristics of a MoS_2_ FET that has been irradiated with Ar^+^ ions with a kinetic energy of 40 eV at fluences of up to 5 x 10^13^ ions/cm^2^. (b) The change in charge carrier concentration was derived from the change in the threshold voltage. At low fluences, the device appears more n-doped, at higher fluences the electron density in MoS_2_ decreases again. Images courtesy of Leon Daniel and Leon Klieve (AG Schleberger), University of Duisburg-Essen.

The radiation tolerance of 2D materials and 2D-based electronic devices has been widely discussed in the literature, see, for example, [228]. With respect to devices, ion irradiation is equally powerful, as it allows for post-fabrication property tuning [229–230]. Early work emphasized the radiation tolerance of FETs based on 2D materials [231]. Indeed, 2D FETs, particularly those with graphene channels, can sustain significant damage while remaining operational. However, one must consider that conventional Si-based MOSFETs use ultrathin dielectrics (a few nanometers). In such devices, the critical fluence before catastrophic gate breakdown can be up to five orders of magnitude lower [232]. In contrast, 2D FETs typically employ much thicker oxides (≈300 nm), which explains their higher fluence tolerance. This interpretation is further supported by recent results on FinFETs, whose narrow channels and gate-all-around architecture confer radiation hardness comparable to that of 2D and CNT FETs [233–234]. Ion irradiation alters electrical performance via several mechanisms, namely, (i) introduction of defects in the channel or dielectric, (ii) modification of the contacts, and (iii) removal of adsorbates and lithography residues. Figure 15 shows an example. The interplay of these effects complicates mechanistic identification. Particularly promising, however, is the ability to tailor trap states responsible for hysteresis in *I*–*V* curves of 2D FETs. This opens pathways not only for conventional optoelectronics but also for novel device concepts such as neuromorphic vision [235] and reservoir computing [236].

### What are the sources for e-beam damage in the transmission electron microscope, and how important are they for different 2D materials?

The three sample-degrading mechanisms in the electron microscope, knock-on effects, inelastic scattering, and chemical etching [237–238], are fundamentally governed by the four parameters electron energy, electronic properties of the sample, sample temperature, and residual molecules in the microscope vacuum. It was shown early that electron irradiation-induced defect creation in graphene in ultrahigh vacuum can be sufficiently well described by the knock-on damage mechanism [239–242], which can be modeled with reasonable accuracy by taking lattice vibrations into account [243]. While increased sample temperature leads to increased probability of damage, it can also hinder its detection due to vacancy [244] and adatom migration [245–246] followed by their annihilation. In contrast to excellent electronic conductors, such as graphene, in semiconducting and insulating materials the knock-on damage mechanism is not sufficient to explain the experimental observations [242,247]. Instead, inelastic scattering processes such as ionization and electronic excitations have to be taken into account [247–250]. Despite the recent efforts to describe this process by combining inelastic and elastic scattering into a single model, so far no universal description has been presented that would adequately describe the experimental data from first principles.

An additional complication is presented by the often unknown influences on the damage creation process arising from sample quality and residual vacuum in the microscope. For example, when graphene is exposed to a relatively low oxygen partial pressure of 3 × 10^−8^ mbar [251], even low-energy electron irradiation, below what is required to displace under-coordinated carbon atoms from a graphene edge, is sufficient to grow pores starting from point defects. This pressure is below what is typical for TEMs with a side-entry sample holder that seals the objective area vacuum with a single O-ring; hence, in many experiments, the roles of pure electron irradiation and electron irradiation-assisted chemical etching can not be distinguished. In comparison, in ultrahigh vacuum, the pore growth becomes negligible at electron energy of 60 keV. The presence of oxygen has also an influence on the structure of the graphene edges [251]: Armchair edges dominate in ultrahigh vacuum, whereas both zigzag and armchair edges are observed during oxygen-mediated etching.

Such chemical effects are not restricted to graphene. It has been known for nearly two decades that triangular pores grow in h-BN during TEM imaging [30,252–253]. However, it was only recently shown that this only occurs in the presence of a low-pressure oxygen atmosphere and that, in ultrahigh vacuum, the pores have a circular shape [254]. This indicates that the displacement cross section of B and N atoms at a h-BN edge are similar enough to cause their displacement at nearly the same probability, at least under 60 and 80 keV electron irradiation, similarly to the observations for the pristine lattice [250]. It has also been shown that a low-pressure oxygen atmosphere accelerates pore growth in the oxidation-sensitive MoTe_2_, whereas, in MoS_2_, it plays a negligible role [255], highlighting that such processes depend on the chemical properties of the material. Finally, similar chemical effects can also occur due to surface contaminations of the sample, regardless of the microscope vacuum. This is due to oxygen, and possibly other reactive species, from the contamination released by the electron irradiation during imaging [256]. It has also been suggested that adatoms (e.g., H and C) present on the surface of 2D materials, can also give rise to additional damage by weakening the in-plane covalent bonds, which, in turn, leads to a decrease in the knock-on damage threshold energy [257].

Due to these complications, accurately quantifying electron-beam damage is challenging. Doing this properly requires controlling, or at least taking into account, all experimental parameters that may have an influence on the relevant processes. For example, chemical effects can be reduced by using atomically clean samples or sample areas far away from contamination and by carrying the experiments out in ultrahigh vacuum (pressure of at least low 10^−9^ mbar or lower). To elucidate the roles of inelastic and elastic damage pathways, experiments must be carried out at different electron energies since their energy-dependences are opposite (inelastic damage becoming more probably at low energies and elastic damage at high energies). Additionally, methodological advances are necessary to reduce the electron fluence required for determination of the atomic structure. This will allow for both quantitative measurements of the damage and damage-free high-resolution imaging. Several approaches can be combined to achieve this goal, such as employing direct-electron detectors, automated and fast scanning, phase reconstruction techniques [258], sparse sampling [259], machine learning-based image recognition techniques [260], and ptychography [261]. This becomes even more important when atomic-scale (S)TEM characterization is applied to materials such as metal-organic frameworks [262] or organic materials, which are even more prone to electron-irradiation damage than traditional 2D materials.

### Can atomistic simulations of the response of supported 2D materials to He ion irradiation in the helium ion microscope be carried out?

Helium ion microscopy (HIM) makes it possible to focus the ion beam on a sub-nanometer area [263] and, thus, to not only get information on the morphology of the sample, but also to create defects with high spatial resolution. For example, the optoelectronic properties of devices assembled from 2D TMDs were modified in a controllable manner [264–266] by defect production using HIM. Free-standing nanoribbons of MoS_2_ have been fabricated [265], along with memristors [267]. Single-photon emitters have been realized in TMDs [268–270] and h-BN [119] by creating defects using HIM, just to mention a few.

**Figure 16:**
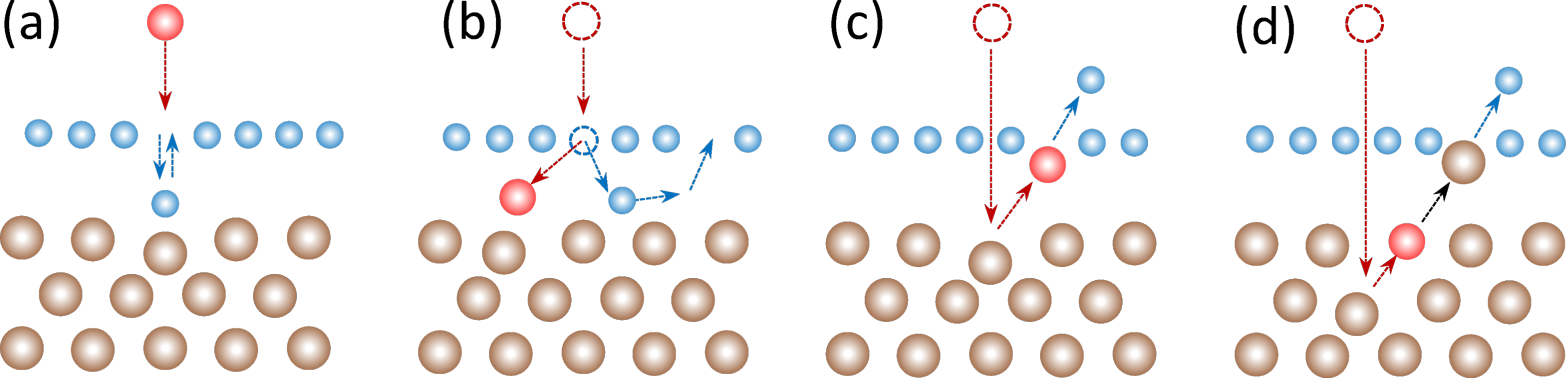
Schematic illustration of the effects of the substrate on defect production in a supported 2D material under ion irradiation. The substrate atoms are colored in brown, the 2D target atom in blue, and the impinging ion is represented as the red circle (filled or empty). The recoil atom sputtered from the 2D material can be reflected by the substrate and immediately be incorporated into the atomic network (a) or diffuse between the 2D material and the substrate and fill a pre-existing vacancy (b). These processes reduce the amount of damage in the irradiated 2D material. The ion can also be backscattered by the substrate (c) or sputter a substrate atom, which in turn displaces an atom from the 2D target. Processes (c) and (d) increase the number of defects in the 2D material. Figure 16 was adapted from [279] (“Simulations of the response of supported 2D materials to ion irradiation with explicit account for the atomic structure of the substrate”, © 2025 M. Jain et al., published by the Royal Society of Chemistry, distributed under the terms of the Creative Commons Attribution 3.0 Unported Licence, http://creativecommons.org/licenses/by/3.0/).

In most of the ion bombardment experiments, the irradiated 2D materials were on a substrate, although free-standing (e.g., deposited on a TEM grid) 2D materials have also been studied. A recent development is an experiment where 2D materials treated with a He-ion beam are transferred to a second substrate before they are analyzed further [119]. Theory predicts [271–274] that the response of the supported 2D materials to ion bombardment can be strongly affected by the presence of a substrate, and the experiments [16,275–278] confirm this. Indeed, depending on ion energy, its charge state and mass, a smaller number of defects can be produced as the atoms displaced from the 2D target may be stopped by the substrate and immediately annihilate with the parent vacancies. The opposite is also possible: The backscattered ions and atoms sputtered from the substrate can enhance defect production, as schematically illustrated in Figure 16.

In HIM, the typical ion energies are in the range of 20–30 keV, which creates enormous computational challenges (even for analytical potential MD simulations) for quantitative prediction of the probabilities of defect creation in the supported 2D materials. The problem is that the cross section for atom displacement is very small so that the ranges of ions are rather big (over 100 nm), while ions backscattered from the atoms deep in the substrate can still reach the surface and create additional damage. Most ions (reaching 99%) do not produce any damage in the 2D material directly; they just go through. Thus, thousands of simulations with various impact points are required to collect the statistics. A work-around was recently suggested [279], namely, using a “special” mesh of impact points, where the points are predominantly sampled in the vicinity of the target atoms, followed by the re-scaling of the outcomes of the simulations as a ratio of the areas near the atoms to the total area. This approach seems to improve the convergence of the results for MoS_2_ on a SiO_2_ substrate, but more extensive simulations and benchmarking are required to validate this approach.

Another problem is to account for in situ annealing of defects at the interface, that is, the annihilation of atoms sputtered from the 2D material and located between the 2D system and the substrate with vacancies. The annealing occurs on a macroscopic time scale; hence, even if the force model used (normally analytical potentials) describes correctly the migration barriers, molecular dynamics simulations are not realistic in practice, as they cannot reach macroscopically long time scales. Stimulated annealing (i.e., rising the temperature) cannot be used as the 2D material can simply detach from the substrate. Kinetic Monte-Carlo simulations with the barriers derived from first-principles calculations can be a solution in this case.

### Can machine learning be used (in practice) in the simulations of defects and ion/electron irradiation?

There is intense interest in the application of machine learning interatomic potentials for both static and dynamic simulations of defects in 2D materials. As for static calculations, MLIPs should, in principle, be able to reproduce DFT levels of precision in defect structure and formation energies, provided that the training set includes defect configurations, with significant gain in computational speed. It is tempting to revisit problems previously tackled using conventional empirical interatomic potentials in order to improve their accuracy. We are at early days, however, with limited available ML multicomponent potentials.

The situation is more complicated in the dynamic calculations of defect production upon impacts of energetic particles. Universal MLIPs (uMLIPs) are trained around equilibrium structures, either from structural relaxation trajectories or MD simulations at moderate temperatures and pressures. Such uMLIPs are expected to work poorly out of the box for simulating irradiation effects, where interionic distances can be very small even in comparison to high-pressure experiments and which usually involve bond breaking and formation. Even for MLIPs trained for a specfic material/system, irradiation simulations are challenging. First, given the sharp potential energy surface at small distances, it will be not straightforward to develop a model that can concurrently treat the equilibrium regions and short-distance regions of the potential energy surface at high accuracy. A possible solution to this is to complement (e.g., by delta-learning) standard MLIPs with models for the empirical models for short-range interactions. Second, the trajectories of irradiated atoms/ions can cover large portions of the phase space, including all different ways to break/form bonds. Thus, the strategy for training data collection becomes critical. Alternatively, one could employ models that provide error/confidence estimates and run the irradiation simulations within active-learning frameworks, using first-principles calculations whenever confidence is low. Third, the models are trained using electronic structures evaluated at 0 K or at most one fixed temperature. That is, they cannot account for different electronic temperatures in different parts of the sample and its coupling to ion dynamics.

In the context of defect–light interactions, one of the most important observables is the photoluminescence (PL) spectrum. When a point defect has localized states inside the bandgap of the host material, transitions between these states, or between the localized states and the delocalized bands of the host can lead to characteristic emission features below the onset of band-to-band transitions. The shape of the PL spectrum is governed by the electron–phonon coupling. Because the latter is sensitive to the chemical identity and detailed atomic structure of the defect center, the PL lineshape constitutes a unique fingerprint essential for identifying the microscopic nature of defects. The calculation of point defect PL spectra from first principles is a rather demanding task. In its most widely used implementation, it requires all vibrational modes of the defect supercell [280]. The latter typically contains several hundreds of atoms; conventional phonon calculations thus require to perform thousands of DFT calculations for a large supercell. The recently introduced uMLIPs based on message-passing neural networks [281–286] have shown remarkable capabilities for several standard tasks such as structure optimization, molecular dynamics, and the calculation of phonons in pristine crystals [287–289]. Recently, it was shown that these uMLIPs (in particular the MatterSim potential [290]) can also be used to obtain the phonons of defect structures with only a small reduction in accuracy. As the MLIPs are orders of magnitude faster than DFT, this completely removes the phonon bottleneck of the PL calculations [115]. In [115], a systematic comparison of PL spectra obtained with phonons from DFT and MLIPs was performed for 791 crystal point defects [291]. Overall, very good agreement was found for the PL lineshape across defects with different charge and magnetic states. The agreement was particularly good for defects with weaker electron–phonon coupling (smaller Huang–Rhys factors), and significant disagreement between DFT and MLIPs was found only in a few cases.

The work by Sharma et al. [115] found that it is essential to relax the defect structure with the MLIP, starting from the DFT-relaxed configuration, prior to calculating the phonons. Using the MLIP to relax the defect structure from scratch was not successful. This shows that there is still room for improvement of the MLIPs in defect modelling; this is most likely because the employed training structures were not sufficiently diverse and/or relevant. Moreover, it should be noted that the calculation of PL spectra, in addition to the ground-state phonons, also involves a relaxation of the defect structure on the excited potential energy surface. This step must be performed using constrained-DFT or a similar methodology as current MLIPs are limited to the electronic ground state. The development of machine learning potentials that would be capable of describing excited-state dynamics is an interesting, yet highly challenging, problem [292].

### How far can we go with machine learning potentials for 2D materials, given their often extreme anisotropy in properties?

The possibility to apply MLIPs for defect modelling is related to a more general question of the applicability of this class of potentials to the simulations of 2D materials. It is not clear a priori whether MLIPs will be able to reproduce the highly anisotropic properties of 2D materials. Single-layer, bilayer, and few-layer materials exhibit substantial structural variations compared to their bulk counterparts. The structural properties of these systems differ from one another, and all few-layered 2D materials show marked distinctions from their bulk forms. This phenomenon is particularly pronounced in 2D materials composed of multiple chemical elements, where local chemical environments heavily influence structural characteristics. For example, graphene maintains a homogeneous carbon distribution within a perfectly flat planar structure, while single-layer C_3_N_4_ exhibits complex buckling due to the distinct chemical properties of carbon and nitrogen atoms (e.g., differences in electronegativity). This buckling structure is layer-dependent and directly affects the resultant electronic configuration, thereby, electronic, optical, and other physical properties. The complexity of local chemistry and structure becomes especially significant in single- and few-layered 2D materials, creating fundamental distinctions from bulk materials.

The mechanical and energetic properties of graphite are the most anisotropic of all materials. While intralayer sigma- and pi-bonding is extremely strong, resulting in an elastic constant C_11_ of over 1000 GPa, the interlayer bonding is a mixture of van der Waals forces and weak electronic delocalization [293], resulting in an interlayer shear elastic constant C_44_ of less than 10 GPa, see Table 1. DFT calculations with empirical dispersion corrections can correctly reproduce this range; however, most conventional empirical potentials cannot, with some exceptions such as the Heggie–Cousins-modified Keating potential developed specifically for this task [294]. A summary table of experimental and calculated elastic constants of graphite is given in Table 1.

**Table 1:** Calculated and experimental elastic constants for graphite. AIREBO, REBO, ACE-C(D2), and ACE-CH(D3) values (marked with *) were generated for this study, and the references indicate the source article for the given potential. All other values are from literature as referenced.

	*C*_11_ [GPa]	*C*_12_ [GPa]	*C*_33_ [GPa]	*C*_44_ [GPa]	*C*_13_ [GPa]

**Experiment**					
ultrasonic, sonic resonance, static testing^a^ [295]	1060 ± 20	180 ± 20	36.5 ± 1.0	0.18–0.35	15 ± 5
pressure derivative from X-ray data^b^ [296]	–	–	–	–	22 ± 2
surface Brillouin scattering^a^ [294]	–	–	–	5.05 ± 0.35	–
**Density functional theory**					
GGA [297]	1079	217	42.2	3.9	−0.5
LDA [297]	1118	235	29.5	4.5	−2.8
**Empirical potentials**					
modified Keating model [294]	1060	180	36.5	5.0	7.9
AIREBO* LJ [298]	960	348	40	0.25	0.25
AIREBO* Morse [299–300]	978	352	36	0.28	0.28
REBO* [301]	843	325	≈0	≈0	≈0
**Machine learning interatomic potentials**					
ACE-C(D2)* [302]	1016	159	35	7.44	−1.7
ACE-CH(D3)* [303]	1287	404	153	7.28	51.41
GAP20^c^ [304]	1022	210	110	35	24

^a^Highly ordered pyrolytic graphite. ^b^Polycrystalline graphite. ^c^Values from [301].

The machine learning potentials quoted here have all been fitted to GGA calculations and, hence, cannot be expected to produce better match to experiment than GGA. Of the carbon MLIPs, the atomic cluster expansion (ACE) potential is of particular interest (MLIPs for carbon materials were recently reviewed in [305]). This shows the importance of the training sets. There is significant error in the interlayer C_44_ constant for a ACE-CH potential developed by Willman et al. [303] and trained on 1435 high-precision snapshots from DFT MD simulations. However there is remarkably accurate reproduction of all GGA-calculated elastic constants for a pure carbon ACE potential of Qamar et al. [302] trained on 17293 systems including structures with different interlayer stacking.

**Figure 17:**
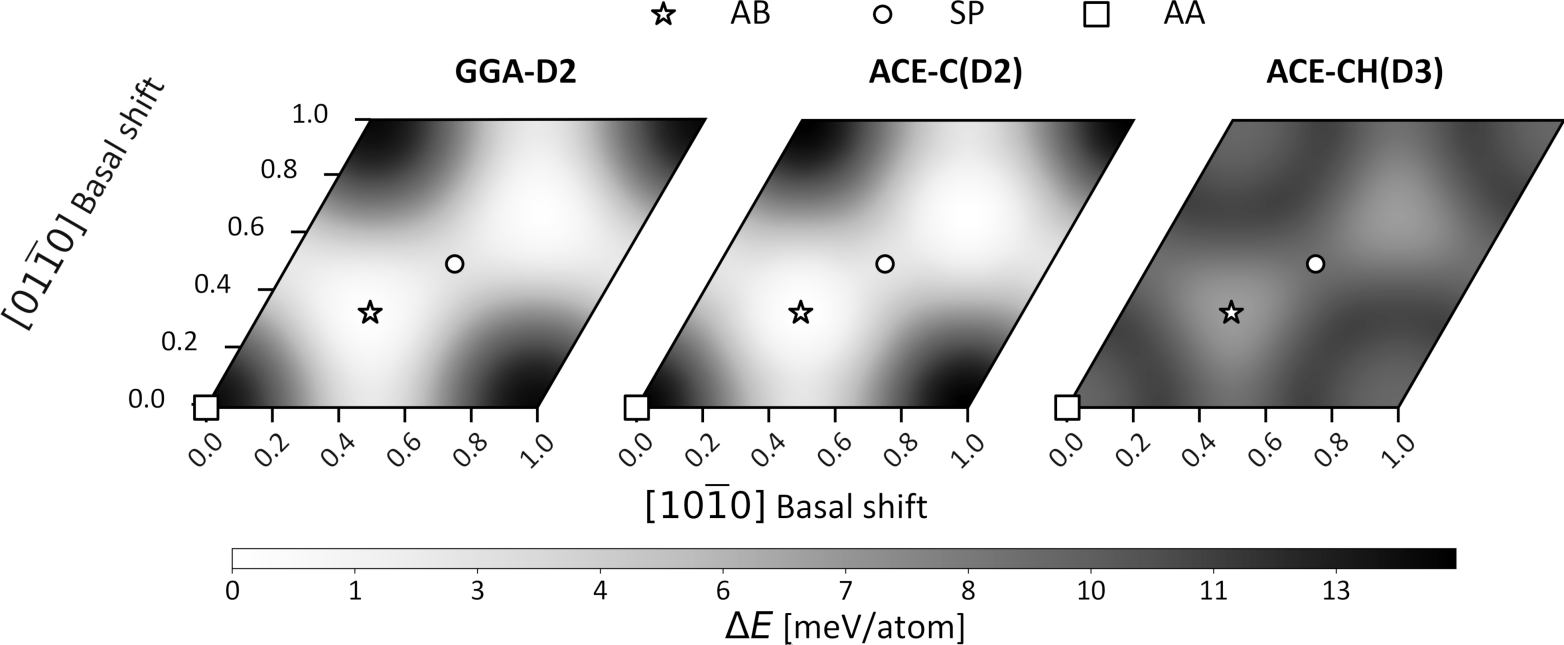
Energy surfaces for different graphitic stackings, calculated using (from left to right): GGA-D2, ACE-C(D2), and ACE-CH(D3). Energies are given relative to the AB values for each method, for a periodic two-layer unit cell (maps generated following the approach in [306]). The left and center maps were reproduced from [306], (© 2026 G. R. Francas et al., published by Elsevier Ltd., distributed under the terms of the Creative Commons Attribution 4.0 International License, https://creativecommons.org/licenses/by/4.0).

Figure 17 shows translational maps of graphitic interlayer stacking energy [306]. Unit cells containing two graphene layers, the first held fixed, and the second translated by fractions of the unit cell vectors. The in-plane lattice coordinates of the second layer are held fixed for each point, and the interlayer spacing are allowed to vary. Plots include ACE-C(D2) and ACE-CH(D3) calculations, and a DFT GGA-D2 calculation for comparison since both ACE MLIPs have been fitted to this level of theory (ACE-CH(D3) fitting to GGA-D3). This is a difficult test that the majority of classical interatomic carbon potentials cannot reproduce, with the exception of potentials developed specifically with this in mind, such as the Kolmogorov–Crespy potential [307]. While both ACE potentials successfully reproduce the global minimum for AB-stacked graphite, only the ACE carbon potential correctly reproduces the whole DFT mapping, including the unstable AA-stacked region. This can be directly attributed to the wide-ranging choice of training sets. Thus, the extreme anisotropy of 2D materials does not appear to be a fundamental stumbling block for MLIPs, but it highlights the importance of appropriate training sets that cover the entire configuration space of both the intra- and interlayer bonding including unstable configurations far from equilibrium. We note that this conclusion will also be important as MLIPs see increasing application in kinetic modelling and dynamic simulations of defect formation and migration. The use of molecular dynamics trajectories as training sets for such potentials will likely not be sufficient since the majority of snapshots will lie in energetic valleys, leading to inaccurate reproduction of saddle point structures and energetics.

The future of defect modelling in layered materials with MLIPs nonetheless appears bright, and the speed gain over DFT calculations opens up a range of previously inaccessible problem types. One example is 1D defects such as dislocations. These are difficult to simulate using traditional methodologies due to their combination of local bonding distortions at the core and long-range strain fields (which decay as 1/*r*) with associated stacking mismatch, requiring prohibitively large systems to minimize dipolar and quadrupolar interactions between neighboring cells. MLIPs have been applied successfully to model dislocations in metals [308], and first attempts at modelling screw dislocations in graphite are promising [306].

## Conclusion

In this article some (but definitely not all) challenges and open questions relevant to the physics and chemistry of defects in 2D materials have been addressed, following the discussion of these issues at the symposium “Defect-mediated engineering of nanomaterials for energy and quantum applications” organized by the Beilstein-Institut [38]. Specifically, the challenges regarding the characterization techniques capable of defect detection and quantification and the defect-mediated engineering of the properties of 2D materials using beams of energetic particles, as well as open questions in the theoretical description of defective 2D materials have been addressed. The clear statements of the problems, open questions, suggested solutions, further ideas, and related discussions should not only attract the attention of the scientific community to the unresolved issues, but also accelerate the progress in the vibrant field of defect-mediated engineering of the properties of 2D materials.

## Data Availability

Data sharing is not applicable as no new data was generated or analyzed in this study.

## Authors

Arkady V. Krasheninnikov^1^, Matthias Batzill^2^, Anouar-Akacha Delenda^3^, Marija Drndić^4^, Chris Ewels^3^, Katharina J. Franke^5^, Mahdi Ghorbani-Asl^1^, Alexander Holleitner^6,7,8^, Ado Jorio^9,10^, Ute Kaiser^11^, Daria Kieczka^12^, Hannu-Pekka Komsa^13^, Jani Kotakoski^14^, Manuel Längle^14^, David Lamprecht^14^, Yun Liu^15^, Steven G. Louie^16,17^, Janina Maultzsch^18^, Thomas Michely^19^, Katherine Milton^12^, Anna Niggas^20^, Hanako Okuno^21^, Joshua A. Robinson^22^, Marika Schleberger^23^, Bruno Schuler^24^, Alexander Shluger^12^, Kazu Suenaga^25,26^, Kristian S. Thygesen^27^, Richard A. Wilhelm^20^, E. Harriet Åhlgren^14,28,29^ and Carla Bittencourt^30^

## Addresses

^1^Institute of Ion Beam Physics and Materials Research, Helmholtz-Zentrum Dresden-Rossendorf, 01328 Dresden, Germany, ^2^Department of Physics, University of South Florida, Tampa, FL 33620, USA, ^3^Institut des Matériaux de Nantes Jean Rouxel (IMN), UMR 6502 CNRS, Nantes University, 44322 Nantes, France, ^4^Department of Physics and Astronomy, University of Pennsylvania, Philadelphia, PA 19104, USA, ^5^Fachbereich Physik and Halle–Berlin–Regensburg Cluster of Excellence CCE, Freie Universität Berlin, 14195 Berlin, Germany, ^6^Munich Center for Quantum Science and Technology (MCQST), 80799 Munich, Germany, ^7^TUM International Graduate School of Science andEngineering (IGSSE), 85748 Garching, Germany, ^8^Walter Schottky Institute, Technical University of Munich, 85748 Garching, Germany,^9^Departamento de Física, Universidade Federal de Minas Gerais, Belo Horizonte, MG 31270-901, Brazil, ^10^International Iberian Nanotechnology Laboratory, 4715-330 Braga, Portugal, ^11^Electron Microscopy Group of Materials Science, Ulm University, Albert-Einstein-Allee 11, D-89081 Ulm, Germany, ^12^Department of Physics and Astronomy and the London Centre for Nanotechnology, University College London, Gower Street, London WC1E 6BT, U.K., ^13^Microelectronics Research Unit, University of Oulu, 90014 Oulu, Finland, ^14^Faculty of Physics, University of Vienna, 1090 Vienna, Austria, ^15^Research School of Chemistry, Australian National University, Canberra, ACT 2601, Australia, ^16^Department of Physics, University of California, Berkeley, Berkeley, CA, USA, ^17^Materials Sciences Division, Lawrence Berkeley National Laboratory, Berkeley, CA, USA, ^18^Department of Physics, Chair of Experimental Physics, Friedrich-Alexander-Universität Erlangen–Nürnberg, 91058 Erlangen, Germany, ^19^II. Physikalisches Institut, Universität zu Köln, 50937 Köln, Germany, ^20^Institute of Applied Physics, TU Wien, 1040 Vienna, Austria, ^21^Université Grenoble Alpes, CEA, IRIG-MEM, 38000 Grenoble, France, ^22^Department of Materials Science and Engineering, The Pennsylvania State University, University Park, PA 16802, USA, ^23^Faculty of Physics and CENIDE, Universität Duisburg–Essen, 47057 Duisburg, Germany, ^24^nanotech@surfaces laboratory, Empa – Swiss Federal Laboratories for Materials Science and Technology, 8600 Dübendorf, Switzerland, ^25^The Institute of Scientific and Industrial Research (ISIR-SANKEN), Osaka University, Osaka 567-0047, Japan, ^26^Nanomaterials Research Institute, National Institute of Advanced Industrial Science and Technology (AIST), Tsukuba 305-8565, Japan, ^27^CAMD, Computational Atomic-Scale Materials Design, Department of Physics, Technical University of Denmark, 2800 Kgs. Lyngby, Denmark, ^28^Department of Physics, University of Helsinki, Pietari Kalmin katu 2, 00560 Helsinki, Finland, ^29^Department of Physics and Astronomy, Ångström Laboratory, Uppsala University, Box 516, SE-751 20 Uppsala, Sweden and ^30^Chimie des Interaction Plasma surface, Research Institute for Materials Science and Engineering, Université de Mons, 7000 Mons, Belgium
